# Micro‐Environment Programmable Quinoline COFs for High‐Performance Photocatalytic H_2_O_2_ Generation and Benzylamine Coupling

**DOI:** 10.1002/advs.202505794

**Published:** 2025-07-17

**Authors:** Mengchao Guo, Chao He, Zihe Wu, Yu Tian, Jiani Yang, Yujie Wang, Hao Wu, Jin Yang, Min Xu, Weichao Xue, Chong Cheng, Shuang Li, Changsheng Zhao

**Affiliations:** ^1^ College of Polymer Science and Engineering National Key Laboratory of Advanced Polymer Materials Sichuan University Chengdu 610065 China; ^2^ Macau Institute of Materials Science and Engineering (MIMSE) Faculty of Innovation Engineering Macau University of Science and Technology Taipa Macau SAR 99078 China; ^3^ Department of Chemistry Chemistry Research Laboratory University of Oxford Oxford OX1 3TA United Kingdom; ^4^ College of Chemistry Sichuan University Chengdu 610065 China

**Keywords:** benzylamine coupling, bioinspired materials, covalent organic frameworks, Hammett relationship, photocatalytic H_2_O_2_ production

## Abstract

Photocatalytic H_2_O_2_ synthesis from water and oxygen by covalent organic frameworks (COFs) has attracted much attention currently. However, conventional COFs often suffer from insufficient stability and activity due to the unclear structure‐activity relationship mechanisms. Herein, a series of quinoline‐linked COFs‐R (‐R = ‐OH, ‐OMe, ‐H, ‐Br, ‐CN) synthesized via multi‐component reactions (MCRs) is reported to systematically modulate their pore microenvironments and enhance photocatalytic performance. Experimental results reveal that the electron‐donating capacity of substituents significantly enhances charge separation efficiency, with H_2_O_2_ production activity exhibiting a negative correlation to the Hammett parameters (*σ_p_*) of the ‐R groups. Notably, the COF‐OH and COF‐OMe, bearing the strong electron‐donating group, achieve a remarkable H_2_O_2_ generation rate of 4458 and 4138 µmol g⁻¹ h⁻¹ in the pure water system. Theoretical calculations confirm that substituents optimize the collective donor structure within the π‐conjugated triazine framework, boosting photocatalytic activity. Furthermore, the universal Hammett relationship observed in benzylamine coupling reactions establishes a critical structure‐activity model for rational COF design. This work provides fundamental insights into the microenvironment engineering of COFs for efficient H_2_O_2_ production and advances the development of sustainable photocatalytic materials.

## Graphical abstract

A series of bio‐inspired quinoline‐based covalent organic frameworks (COFs) with varying substituents (‐R) are synthesized via multi‐component reactions. These substituents demonstrated a pronounced structure‐property relationship between their electronic effects (Hammett parameter, *σ_p_*) and the photocatalytic H₂O₂ production rate, as well as the conversion efficiency of benzylamine oxidative coupling reactions.

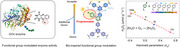

## 1 Introduction

Hydrogen peroxide (H_2_O_2_) is one of the most important oxidants and is widely utilized for many relevant applications in wastewater treatment, paper bleaching, disinfection, chemical synthesis, and also as an alternative source of stored energy.^[^ ^1^, ^2^, ^3^, ^4^ ^]^ Its global annual demand is 4 million tons, which will be increased to an estimated 5.7 million tons by 2027.^[^ ^5^, ^6^ ^]^ Currently, industrial H_2_O_2_ production still relies on the anthraquinone process, which is energy‐intensive and produces hazardous waste.^[^ ^7^ ^]^ Therefore, the development of sustainable, eco‐friendly, and cost‐efficient H_2_O_2_ production technologies is highly demanded. In this context, photocatalytic H_2_O_2_ production from water and oxygen has become a sustainable alternative.^[^ ^8^, ^9^, ^10^ ^]^ Therefore, the development of efficient semiconductors with tunable optical and electronic properties is promising and of great importance in this photocatalyst area.

Recently, the newly emerged organic semiconductors, covalent organic frameworks (COFs) with tunable porous structures, crystalline networks,^[^ ^11^, ^12^, ^13^, ^14^, ^15^ ^]^ and semiconductor properties have attracted considerable attention in the photocatalytic H_2_O_2_ production area.^[^ ^16^, ^17^, ^18^, ^19^, ^20^ ^]^ Thomas's group first reported COFs in the photocatalytic H_2_O_2_ production with the rate of 234.5 µmol h^−1^ g^−1^ in the year 2020, which then attracted considerable attention and followed research (Table S1, Supporting Information).^[^ ^21^ ^]^ For example, the quinoline‐based MeO‐QN‐TA‐COF functionalized with methoxy groups attained a remarkable H₂O₂ production rate of 7384 µmol g⁻¹ h⁻¹ in pure water under blue light irradiation.^[^ ^22^ ^]^ Additionally, the pyridine‐based TBA‐COF exhibited outstanding performance, achieving a high H₂O₂ production rate of 8878 µmol g⁻¹ h⁻¹ in pure water.^[^ ^23^ ^]^ However, most of the early reported COFs show insufficient stability in acidic or alkaline solutions due to the reversibility of covalent bonds in most COFs, which hinders their long‐term application in H_2_O_2_ production. As an example, Cooper and co‐workers use a high‐throughput sonochemical strategy to synthesize 60 kinds of crystalline imine COFs for photocatalytic H_2_O_2_ production.^[^ ^24^ ^]^ Among them, sonoCOF‐F2 shows excellent photocatalytic activity for H_2_O_2_ production, but it still transformed into an amide‐linked COF with reduced crystallinity and loss of electronic conjugation, decreasing the photocatalytic activity after long‐term photocatalytic tests. Most recently, the multi‐component reactions (MCRs) reported by Thomas’ group enable a facile synthetic approach by combining multiple components in one pot to form robust aromatic linkages and thus chemically and temperature‐stable COFs.^[^ ^25^, ^26^, ^27^ ^]^ For example, the one‐pot synthesis of quinoline‐linked COF by using the multicomponent Povarov reaction, which can keep high chemical stability under harsh conditions and produce high amounts of H_2_O_2_ under natural sunlight over a long period (60 h) without loss in catalytic activity. Such an MCR process not only gives a stable aromatic linkage for the synthesized COFs but also brings a simple strategy to introduce versatile site groups in the COF structures.^[^ ^22^, ^28^, ^29^, ^30^, ^31^, ^32^, ^33^, ^34^, ^35^ ^]^

Despite impressive progress in developing various types of COFs and exploring their potential applications, most studies employ empirical explorations, and rational design strategies have rarely been used in designing high photoactive COFs.^[^ ^36^, ^37^, ^38^ ^]^ Looking into the natural enzyme system, most of the highly active and highly selective enzyme catalysis in nature is achieved through the fine protein structure and microenvironment regulation around the enzyme.^[^ ^39^, ^40^ ^]^ For example, the Flavin Adenine Dinucleotide (FAD) cofactor in glucose oxidase, composed of a flavin core and an extended side chain, plays a pivotal role in both structural stabilization and catalytic performance.^[^ ^41^ ^]^ The extended side chain of FAD spatially anchors the flavin core within a hydrophobic pocket formed by surrounding amino acid residues, regulating the electronic properties and substrate selectivity of the flavin core. Therefore, the analysis of the relationship between microenvironment and properties in COF structure is an important way to guide the development and design of high‐performance COFs in the future. Nevertheless, for most of the currently developed COFs, explicit quantitative structure‐activity relationships have not been systematically identified due to the difficulties in synthesizing a series of suitable COF structures for investigation. It is of great importance to synthesize a series of similar COF structures with different functional groups in the pore structure for a comprehensive investigation of the micro‐environment influence on COFs’ photocatalytic activities and analyze their structure‐activity relationships.

**Figure 1:**
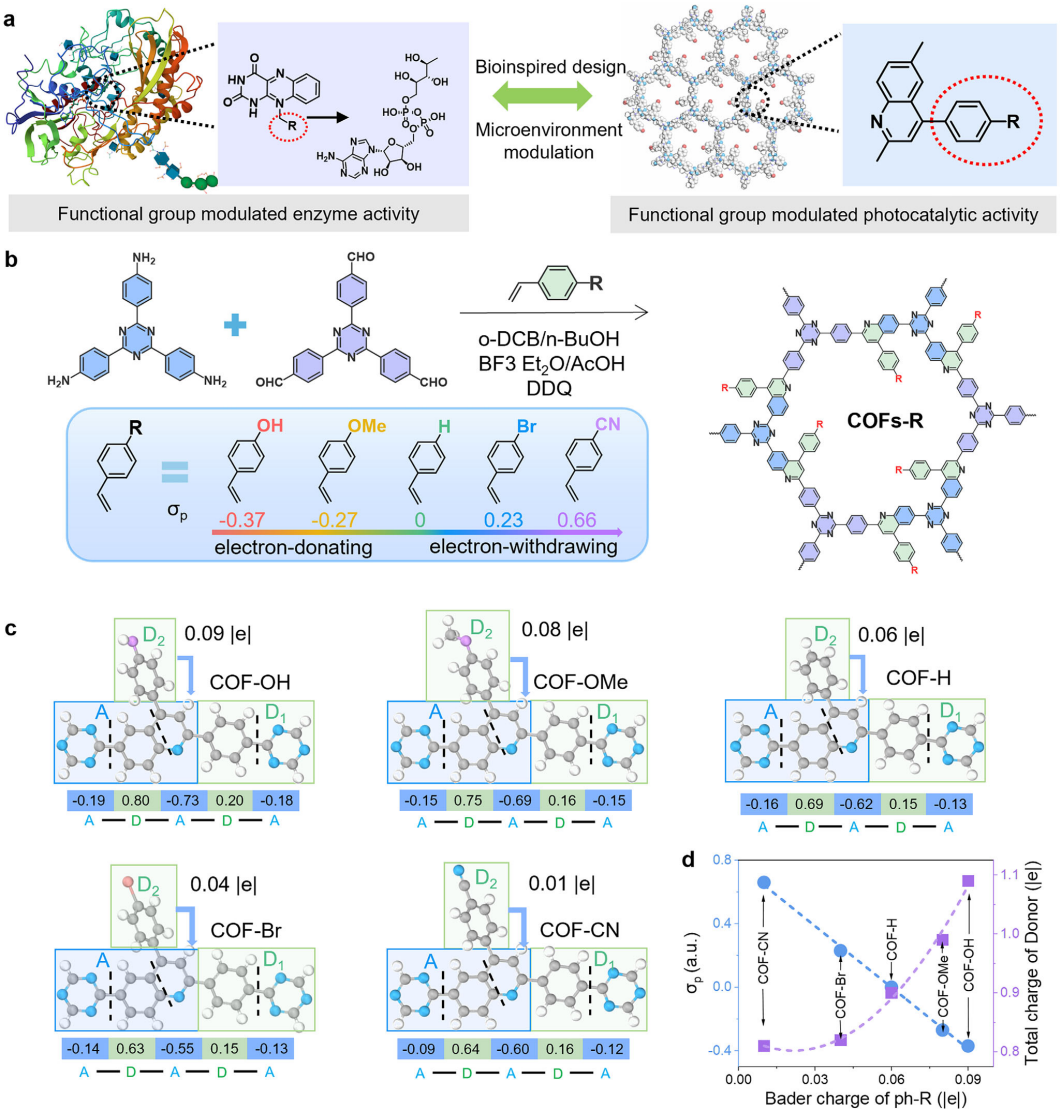
Design of quinoline‐based COFs‐R via MCR synthesis strategy. a) Glucose oxidase‐inspired COF modulation. The protein drawing is based on the crystal structure of an oxidoreductase (PDB ID: 00001cf3). b) Synthesis scheme for COFs‐R (‐R = ‐OH, ‐OMe, ‐H, ‐Br, and ‐CN). c) Bader charge transfer for different substituents. d) The relationship between D‐A structure and Hammett parameters.

Herein, we employed the MCRs strategy and synthesized a series of quinoline‐based COFs with different functional groups, COFs‐R (‐R = ‐OH, ‐OMe, ‐H, ‐Br, and ‐CN). The quinoline backbone shows a similarity with glucose oxidase, whose activity can be well adjusted by the side‐R groups (**Figure** **1**a). The unique structure of COFs‐R with continuous π‐conjugation can ensure excellent electron transfer for photocatalysis. Theoretical calculations indicate that the linker substituents R provide additional donor structures to the COF triazine framework. As the electron‐donating ability of the substituents increases, the COFs exhibit enhanced collective donor structure and improved donor–acceptor (D‐A) separation efficiency. Through systematic investigation of their photocatalytic H_2_O_2_ generation activity, we found that the photocatalytic activity is negatively correlated with the Hammett *σ_p_* values of the linker substituent R in COFs‐R, which is highly related to the electron‐donating capacity of the functional groups. The highest H_2_O_2_ generation activity of the COFs can reach 4458 µmol g^−1^ h^−1^ for the COF‐OH. More importantly, similar Hammett relationships have also been observed in various benzylamine coupling reactions. This will build a feasible bridge for subsequent research into the relationship between the structure and performance of photocatalytic COFs, thereby providing theoretical guidance and an experimental basis for the design and optimization of new photocatalytic materials.

## 2 Results

The 4,4',4''‐(1,3,5‐Triazine‐2,4,6‐triyl) trianiline (TTT) and 4,4',4‐(1,3,5‐triazine‐2,4,6‐triyl) tribenzaldehyde (TTA) are chosen as the two main components for these COFs synthesis to exclude the influence of donor‐acceptor directions. Then, a series of benzene alkenes with different groups is chosen as the third component to yield the corresponding COFs‐R (Figure 1b). The pristine two‐component Im‐COF based on TTT and TTA are synthesized for comparison (Scheme S1, Supporting Information). We employed Hammett *σ_p_* to quantify the electronic distribution imparted by substituents on the quinoline ring.^[^ ^42^ ^]^ When setting the Hammett value for the phenyl group (Ph) to 0, the values for Ph‐OH, Ph‐OMe, Ph‐Br, and Ph‐CN are −0.37, −0.27, 0.23, and 0.66, respectively, indicating a gradual transition from electron‐donating to electron‐withdrawing effects. Bader charge analysis performed by density functional theory (DFT) indicates that the substituents exhibit electron‐donating properties relative to the quinoline core, with their electron‐donating ability negatively correlated with the Hammett *σ_p_* (Figure 1c). As shown in Figure 1d, the Bader charge of these substituents gradually increases from 0.01 |e| for ‐CN to 0.09 |e| for ‐OH, which provides additional donor structures to the triazine covalent organic framework, enhancing its overall donor capability and D‐A separation.

**Figure 2:**
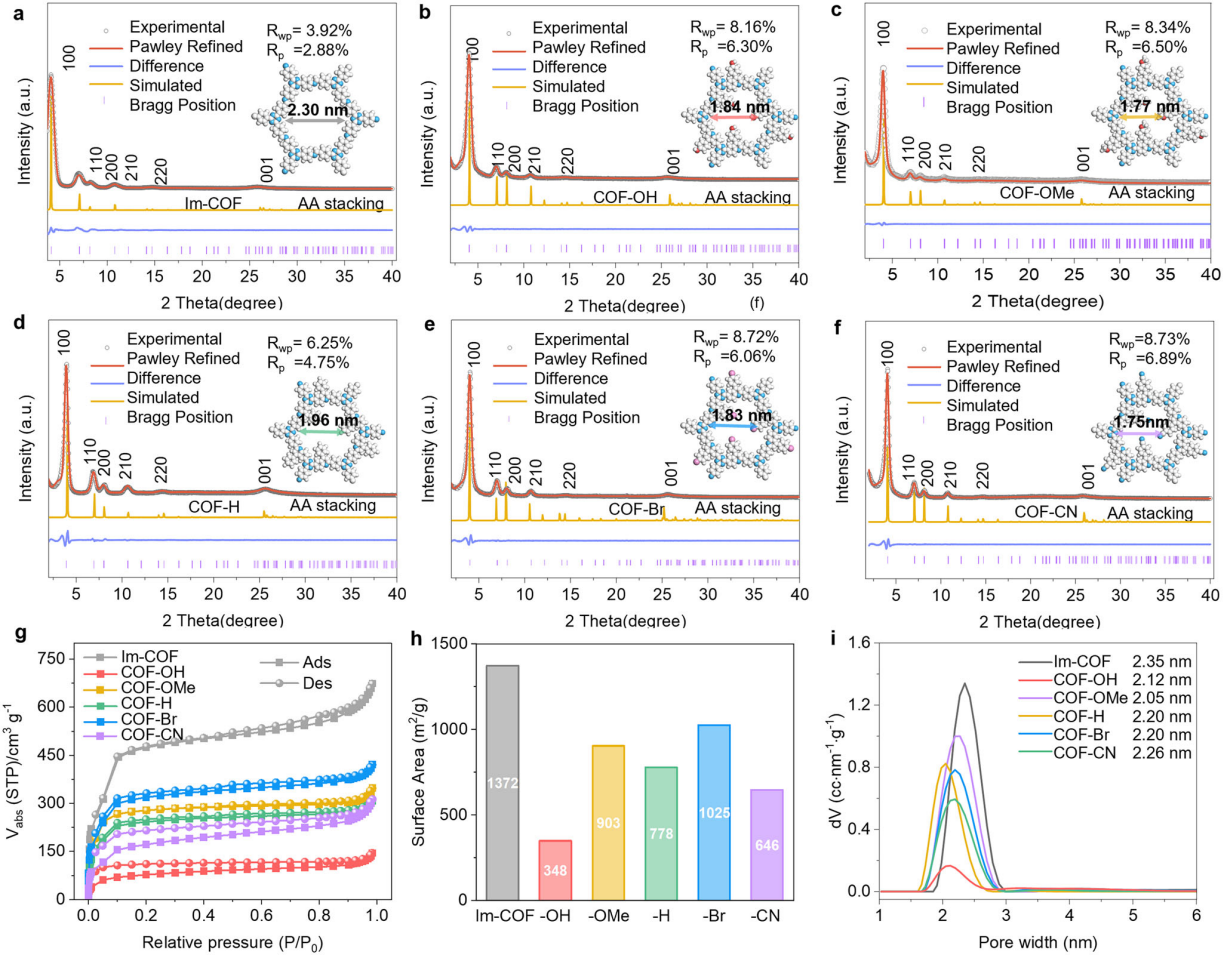
Physical structure properties of photocatalytic COFs. Pawley refined and experimentally obtained PXRD pattern with a minimum difference for hexagonal AA stacking of a) Im‐COF, b) COF‐OH, c) COF‐OMe, d) COF‐H, e) COF‐Br, and f) COF‐CN. g) N_2_ sorption isotherms of the synthesized Im‐COF and COFs‐R at 77K, h) their calculated specific surface area, and i) their calculated pore size distributions.

Powder X‐ray diffraction (PXRD) patterns of the synthesized COFs‐R exhibited well‐defined sharp diffraction peaks similar to those of Im‐COF, indicative of their similar crystallographic structures and high crystallinity. Specifically, the pristine Im‐COF shows the most intense peak at F 2θ, with less intense peaks appearing at ≈7.0°, 8.1°, 10.8°, 14.6°, and 25.9° 2θ, correspondence to the reflections from the (100), (110), (200), (210), (220), and (001) facets, respectively (**Figure** **2**a). The broad and weak reflections observed ≈25° to 26° 2θ correspond to an interlayer distance of ≈3.5 Å, confirming the presence of π–π stacking, which is characteristic of 2D layered structures in both COFs‐R and imine‐based COFs. The simulated PXRD pattern suggested that all COFs matched well with their eclipsed AA layer models (Figure 2a–f; Figures S1–S3, Supporting Information), and further Pawley refinements have determined the unit cell parameters for Im‐COF (a = b = 23.44 Å, c = 3.19 Å, *R_wp_* = 3.92%, and *R_p_* = 2.88%), COF‐OH (a = b = 24.86 Å, c = 3.42 Å, *R_wp_* = 8.16%, and *R_p_* = 6.30%), COF‐OMe (a = b = 24.76 Å, c = 3.40 Å, *R_wp_* = 8.34%, and *R_p_* = 6.50%), COF‐H (a = b = 25.24 Å, c = 3.49 Å, *R_wp_* = 6.25%, and *R_p_* = 4.75%), COF‐Br (a = b = 25.05 Å, c = 3.43 Å, *R_wp_* = 8.72%, and R_p_ = 6.06%), and COF‐CN (a = b = 25.34 Å, c = 3.33 Å, *R_wp_* = 8.73%, and *R_p_* = 6.89%), with α = β = 90°, γ = 120° (Table S2, Supporting Information). All the refinement results exhibited excellent fitting, suggesting that the introduction of these substituents didn't change the fundamental structure of the pristine imine COF (Table S3, Supporting Information). However, slight systematic changes in the unit cell parameters were observed after the introduction of these substituents, which are likely due to changes in the pore environment (Table S4, Supporting Information). Then, the pore structures of all the COFs‐R and Im‐COF were investigated by nitrogen sorption analysis at 77K, where the Im‐COF exhibited a high Brunauer‐Emmett‐Teller (BET) surface area of 1372 m^2^ g^−1^. After the addition of quinoline‐R groups, their BET surface area decreased to 348, 903, 778, 1025, and 648 m^2^ g^−1^ for COF‐OH, COF‐OMe, COF‐H, COF‐Br, and COF‐CN, respectively (Figure 2g,h). It can be noticed that the COF‐OH shows the lowest surface area, which may be due to the strong hydrogen interaction of ‐OH groups in the pore structure, blocking the nitrogen adsorption. The pore size distribution calculated by quenched solid density functional theory reveals a pore size of 2.35 nm for Im‐COF, which is slightly larger than the quinoline‐based COFs (≈2.12, ≈2.05, ≈2.20, ≈2.20, and ≈2.26 nm for COF‐OH, COF‐OMe, COF‐H, COF‐Br, and COF‐CN, respectively). The decrease in surface area and pore size can be a result of the additional functional groups in the pore structures.

**Figure 3:**
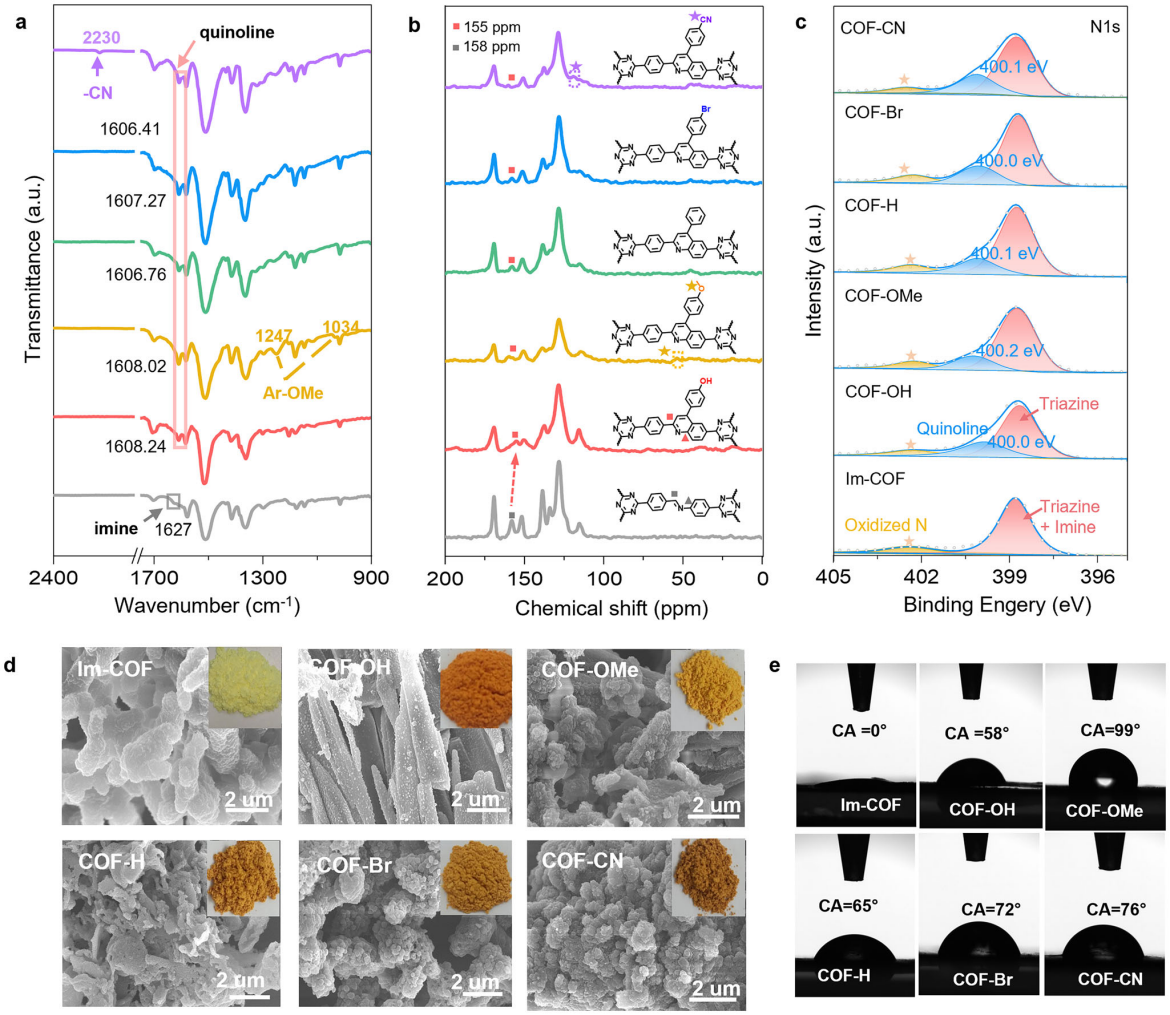
Chemical structure properties of photocatalytic COFs. a) FT‐IR spectra, b) Solid‐state ^13^C NMR spectra, c) N 1s XPS spectra of Im‐COF and COFs‐R (The yellow star at 402.5 eV corresponds to surface oxidation of nitrogen during the treatment process), d) SEM, and e) Water contact angles of water droplets on the pressed pellet of Im‐COF and COFs‐R.

Fourier transform infrared (FT‐IR) spectroscopy and solid‐state ^13^C nuclear magnetic resonance (NMR) spectroscopy were then performed to confirm the bond formation. As shown in **Figure** **3**a, the Im‐COF shows a feature peak at≈1627 cm^−1^ of the imine group,^[^ ^43^ ^]^ while only the pyridyl stretching frequency corresponding to the quinoline‐rings at≈1608 cm^−1^ was observed for all COFs‐R,^[^ ^33^, ^44^, ^45^ ^]^ indicating the successful formation of the quinoline‐ring. Specifically, the –CN stretching vibration of COF‐CN (∼2230 cm⁻¹), and the symmetric and asymmetric –OMe stretching bands in COF‐OMe (1034 and 1247 cm⁻¹, respectively) indicate the successful introduction of the corresponding –R substituents.^[^ ^28^, ^46^ ^]^ The solid‐state ^13^C NMR spectra reveal that the feature carbon peak of ‐C = N‐ bonds at 158 ppm in Im‐COF shifted to 155 ppm, which can be assigned to the signal of carbon in quinoline groups, further confirming the successful formation of the quinoline‐ring in COFs‐R (Figure 3b). The X‐ray photoelectron spectroscopy (XPS) was then conducted to confirm the elemental composition and chemical state of the as‐synthesized Im‐COF and COFs‐R.^[^ ^26^ ^]^ The C, N, and O peaks can be observed in all COFs, while the Br peak is specifically observed in the COF‐Br, indicating the successful introduction of the ‐Br group (Figure S4 and Table S5, Supporting Information). The high‐resolution N1s peak fitting in Figure 3c reveals a clear quinoline N formation for COFs‐R at≈400.5 eV, indicating the successful formation of quinoline linkages (Figure 3c). The morphologies of the Im‐COF and COFs‐R are investigated by scanning electron microscope (SEM) and transmission electron microscope (TEM). As shown by SEM images in Figure 3d, all of the as‐synthesized COFs show similar micro‐sized particle morphology. TEM images of the representative COF‐OMe show the lattice fringes with a distance of 1.85 nm, revealing the good crystallinity of COF‐OMe (Figure S5, Supporting Information). The contact angle on Im‐COF exhibits a contact angle (CA) ≈0°, indicating the hydrophilic property, while changes in contact angles are observed for COF‐H (65°), COF‐OH (58°), COF‐OMe (99°), COF‐Br (72°), and COF‐CN (76°), indicating the hydrophobic property of quino line linked COFs (Figure 3e; Figure S6, Supporting Information).

**Figure 4:**
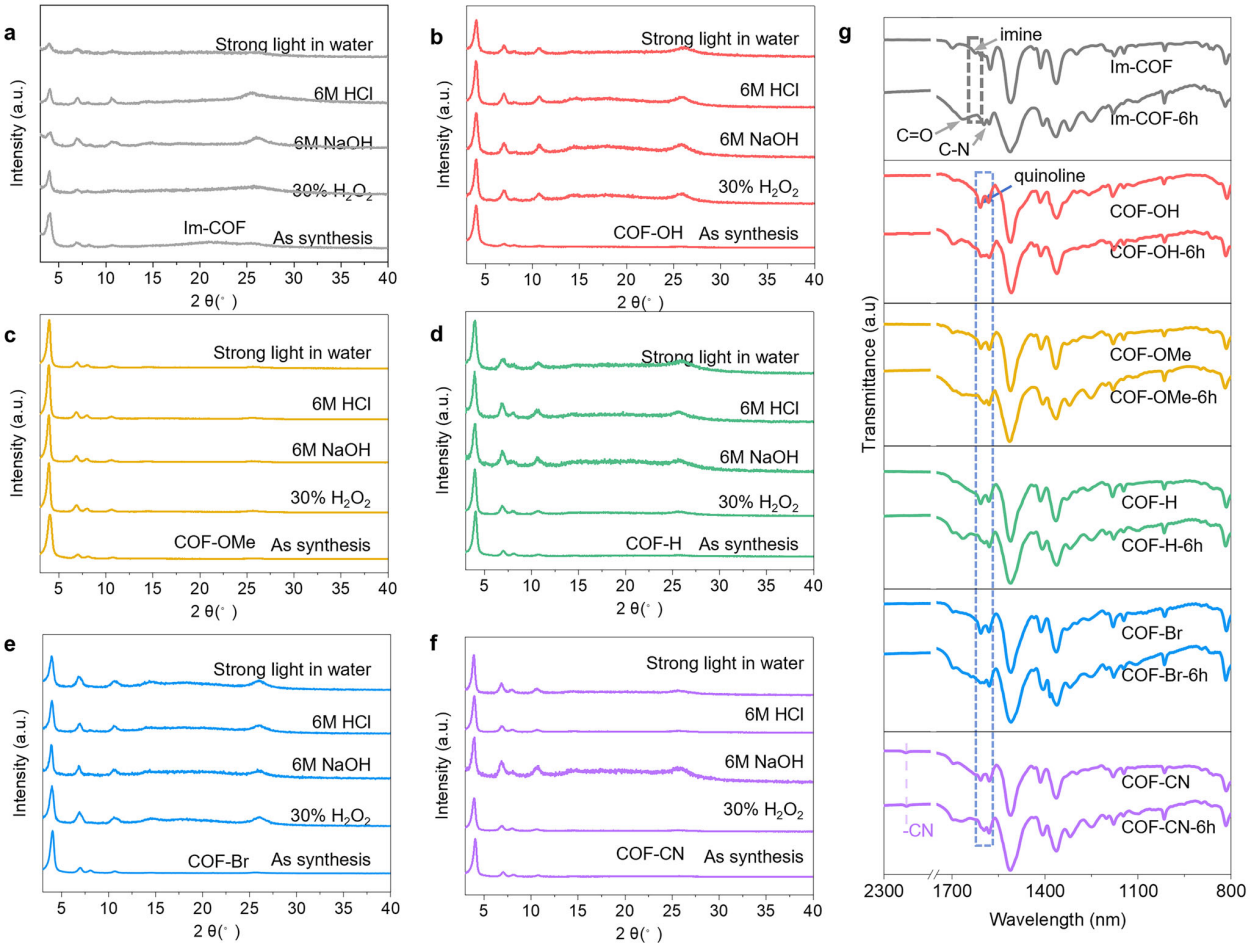
Stability under harsh conditions for photocatalytic COFs. a‐f) PXRD patterns of Im‐COF and COFs‐R for prolonged treatments in strong mineral acid (6 M HCl, 12 h), a strong base (6 M NaOH, 12 h), a strong oxidant (30% H_2_O_2_, 12 h), and strong light (water, 6 h). g) FTIR patterns after 6 h of reaction in pure water.

To assess the chemical stabilities of the modified framework structures, these COFs were subjected to a variety of harsh chemical conditions, including strong mineral acid (6 M HCl, 12 h), strong base (6 M NaOH, 12 h), strong oxidant (30% H_2_O_2_, 12 h) and strong light (water, 6 h). As shown in **Figure** **4**a, a remarkable PXRD peak intensity decrease is observed in these harsh conditions for the Im‐COF. In contrast, all the quinoline‐linked COFs‐R barely show any peak intensity decrease, indicating the strikingly improved chemical stability (Figure 4b–f). The FT‐IR spectra of these COFs before and after photocatalysis for 6 h, the Im‐COF exhibited a pronounced N‐C = O stretching vibration peak, indicating the oxidation of the imine bond to amide bond, while the quinoline group in COFs‐R maintains stable, further indicating their better chemical stability (Figure 4g).

**Figure 5:**
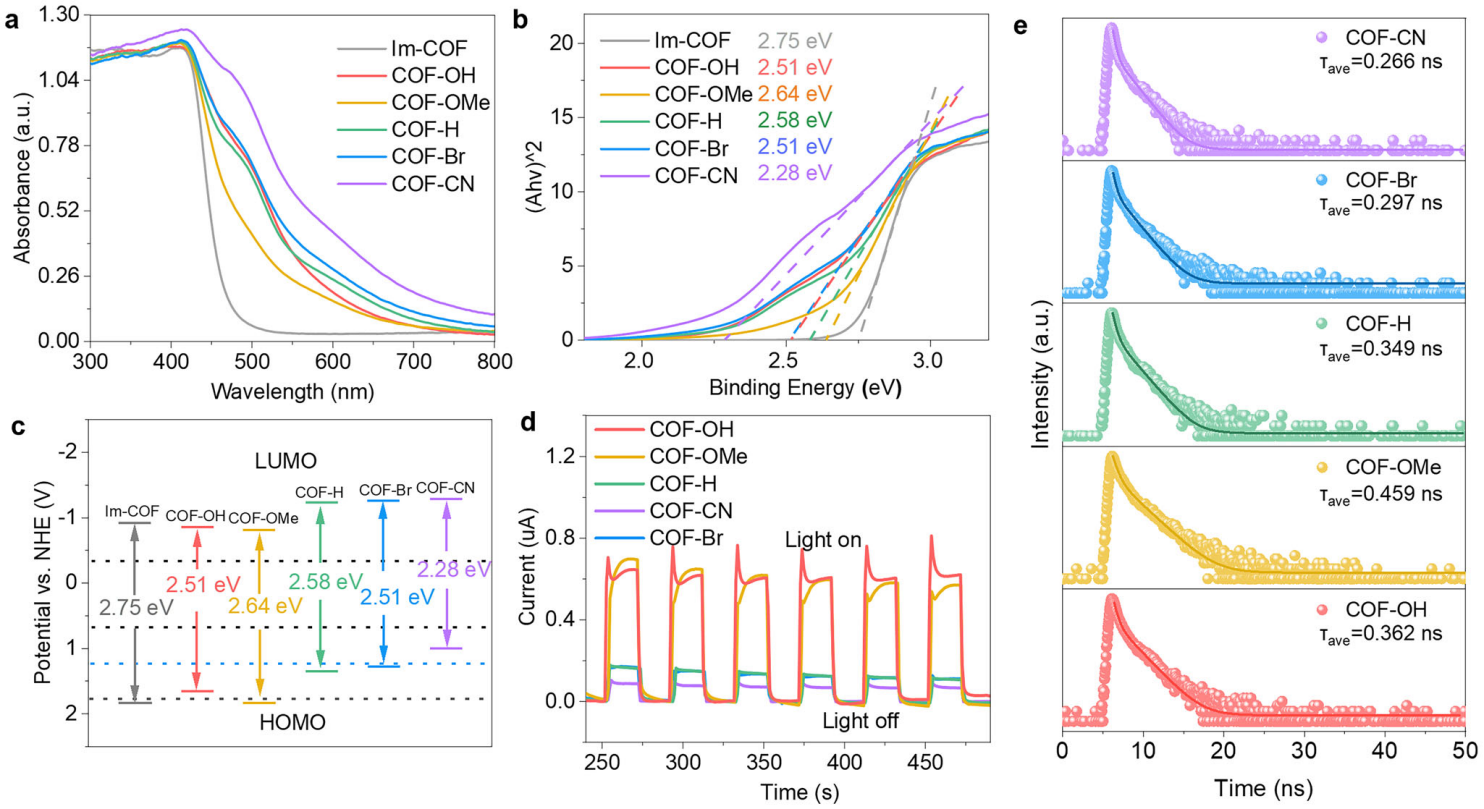
Optical and optoelectronic properties. a) solid‐state UV–vis DRS spectra and b) Kubelka‐Munk‐transformed reflectance spectra of Im‐COF and COFs‐R. c) HOMO and LUMO levels alignments of Im‐COF and COFs‐R. d) Transient photocurrents of COFs‐R measured under the full‐arc spectrum. e) Time‐resolved fluorescence decay curves of COFs‐R.

The photochemical property of the Im‐COF and COFs‐R was first investigated by solid‐state UV–vis diffuse reflectance spectrum (UV‐vis DRS). All of the quinoline‐linked COFs‐R exhibited broader absorption ranges than the Im‐COF owing to their increased π conjugation (**Figure** **5**a). After Kubelka−Munk transforming based on the solid‐state UV–vis DRS results, the corresponding optical bandgaps of COF‐OH, COF‐OMe, COF‐H, COF‐Br, and COF‐CN were calculated to be 2.51, 2.64, 2.58, 2.51, and 2.28 eV, which are all lower than the 2.75 eV for Im‐COF (Figure 5b). The flat‐band potential (FBP) values were measured by the Mott−Schottky tests under different frequencies with a three‐electrode system. The calculated FBP are −1.05, −1.01, −1.44, −1.45, and −1.49 V versus. Ag/AgCl for COF‐OH, COF‐OMe, COF‐H, COF‐Br, and COF‐CN, respectively (Figure S7, Supporting Information), indicating that their conduction bands (CB) based on normal hydrogen electrode (NHE) were −0.85, −0.81, −1.24, −1.25, and −1.29 V, respectively. According to the equation *E_CB_ = E_VB_ − E_g_*, the valence band (VB) values are calculated to be 1.83, 1.66, 1.83, 1.34, 1.26, and 0.99 V versus. NHE for Im‐COF, COF‐OH, COF‐OMe, COF‐H, COF‐Br, and COF‐CN, respectively (Figure 5c). Interestingly, the VB and CB levels of COFs‐R are distinct and highly dependent on their functional groups, where the COFs‐R with electron‐donating groups have higher lowest unoccupied molecular orbital (LUMO) and highest occupied molecular orbital (HOMO) values. The theoretic calculated HOMO and LUMO levels in DFT based on a complete single‐crystal unit cell model show some different results, which might due to the unavoidable defects during materials synthesis (Figures S8 and S9, Supporting Information).

Transient photocurrent response measurements were conducted to investigate their effectiveness toward photocurrent response, electron‐hole separation, and charge‐carrier transfer properties. Continuous and intense photocurrent responses were recorded in all samples from the light switching on/off during a certain interval, where the photocurrent intensity of the electron‐donating group‐substituted COF‐OH and COF‐OMe was significantly enhanced compared to others, indicating an improved separation efficiency of photogenerated electrons and holes within COF‐OH and COF‐OMe (Figure 5d). The results of electrical impedance spectra also indicate that COF‐OH and COF‐OMe have smaller charge transfer resistance, which leads to a faster interface electron transfer speed (Figure S10, Supporting Information). In addition, the average fluorescence lifetime (*τ_a_*) of COF‐OH, COF‐OMe, COF‐H, COF‐Br, and COF‐CN is 0.36, 0.459, 0.349, 0.297, and 0.266 ns, respectively (Figure 5e). The electron‐donating substituted COF‐OH and COF‐OMe have a relatively longer *τ_a_* and stronger oxidative ability, which would facilitate the formation of more reactive oxygen species and increase their H_2_O_2_ yield.

**Figure 6:**
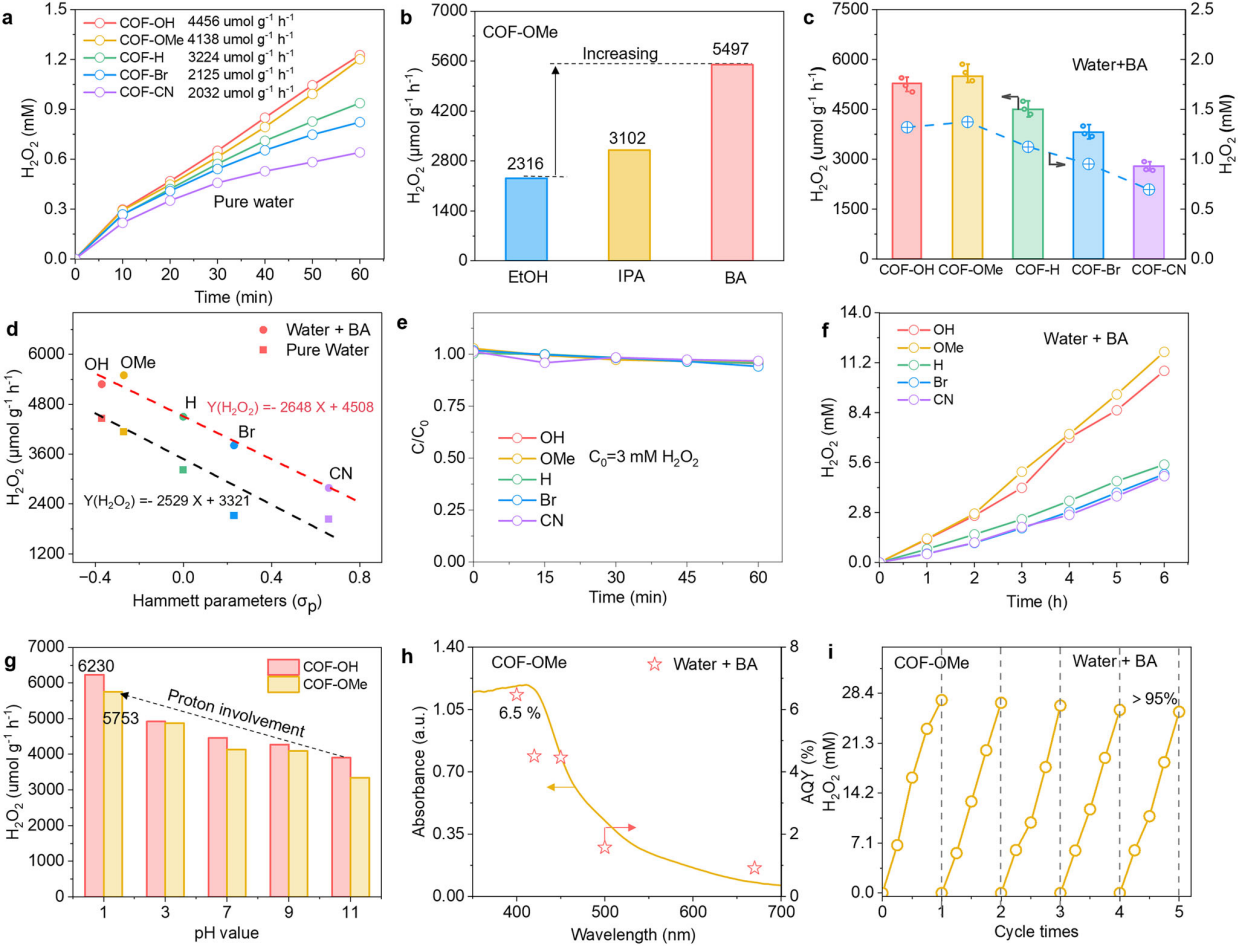
The photocatalytic performance of COFs‐R for H_2_O_2_ production. a) H_2_O_2_ production of COFs‐R in a pure water system. b) H_2_O_2_ production by COF‐OMe with different sacrificial agents (EtOH, IPA, and BA). c) H_2_O_2_ production of COFs‐R in water + BA. d) The relationship between the photocatalytic performance of COFs‐R (Y) and Hammett parameters (X). e) H_2_O_2_ decomposition tests for COFs‐R. f) A long‐term H_2_O_2_ production curve over COFs‐R in water/BA. g) H₂O₂ production of COF‐OH and COF‐OMe in aqueous solution under different pH values. h) Apparent quantum efficiency of COF‐OMe. i) The cyclability of COF‐OMe for H_2_O_2_ photosynthesis in water/BA.

Photocatalytic experiments were then carried out to assess the performance of quinoline‐linked COFs‐R in an O_2_‐saturated water system under visible light (5 mg of COFs in 20 mL water, measured after 1 h at 25 °C and λ>420 nm) (Figures S11 and S12, Supporting Information). As shown in **Figure** **6**a, the H_2_O_2_ production rate of these COFs‐R in pure water was measured, where the COF‐OH and COF‐OMe with strong electron‐donating groups show the best performance with the H_2_O_2_ production rate of 4456 and 4138 µmol g^−1^ h^−1^. In contrast, COF‐H, COF‐Br, and COF‐CN containing weaker electron‐withdrawing groups show lower H_2_O_2_ production rates of 3224, 2125, and 2032 µmol g^−1^ h^−1^, respectively. When ethanol, isopropanol (IPA), and benzyl alcohol (BA) were used as sacrificial agents, the H₂O₂ generation rates of the representative COF‐OMe were measured to be 2316, 3102, and 5497 µmol·g⁻¹·h⁻¹, respectively, where the BA is supposed to be the best sacrificial agent (Figure 6b). The H_2_O_2_ production rate of the COF‐OH, COF‐H, COF‐Br, and COF‐CN can also be improved to 5284, 4500, 3812, and 2788 µmol g^−1^ h^−1^ with BA in the system (Figure 6c), which show a similar trend with the performance in pure water and negatively correlated to Hammett *σ_p_* values of the ‐R group regardless of the presence or absence of a sacrificial agent (Figure 6d).

In addition, photocatalytic H_2_O_2_ decomposition tests under visible‐light irradiation in the presence of COFs‐R were also carried out. As shown in Figure 6e, more than 95% H_2_O_2_ is maintained for all quinoline‐based COFs after irradiation for 1 h, revealing they are inactive toward H_2_O_2_ decomposition under photoirradiation, therefore favoring the continuous photocatalytic H_2_O_2_ production. Continuous H_2_O_2_ production was then tested for 6 h; the production amount of H_2_O_2_ steadily increased over time and reached 10.75 and 11.81 mm for COF‐OH and COF‐OMe, which are much higher than the COF‐H, COF‐Br, and COF‐CN (Figure 6f). The photocatalytic H_2_O_2_ production of COF‐OH and COF‐OMe was further examined under various pH conditions. Notably, at pH 1 without the use of sacrificial reagents, the photocatalytic H_2_O_2_ production rates were 6230 and 5753 µmol g^−1^ h^−1^, respectively (Figure 6g). The enhanced photocatalytic performance of COF‐OH and COF‐OMe with the decreased pH value indicates the participation of protons in the generation of H_2_O_2_. The appearance quantum yields (AQY) of the best‐performed COF‐OMe show a strong dependence on its absorption spectrum and reach 6.5 % at 400 nm within 1 h (Figure 6h). In addition, COF‐OMe exhibited an excellent solar‐to‐chemical energy conversion value of 0.16% with wavelength λ ≥ 400 nm within 1 h (Table S6, Supporting Information). The recycle stability of COF‐OMe was measured by cyclic experiments five times in the water + BA system, where the H_2_O_2_ yield keeps more than 95% after five cycles, indicating its excellent stability (Figure 6i).

**Figure 7:**
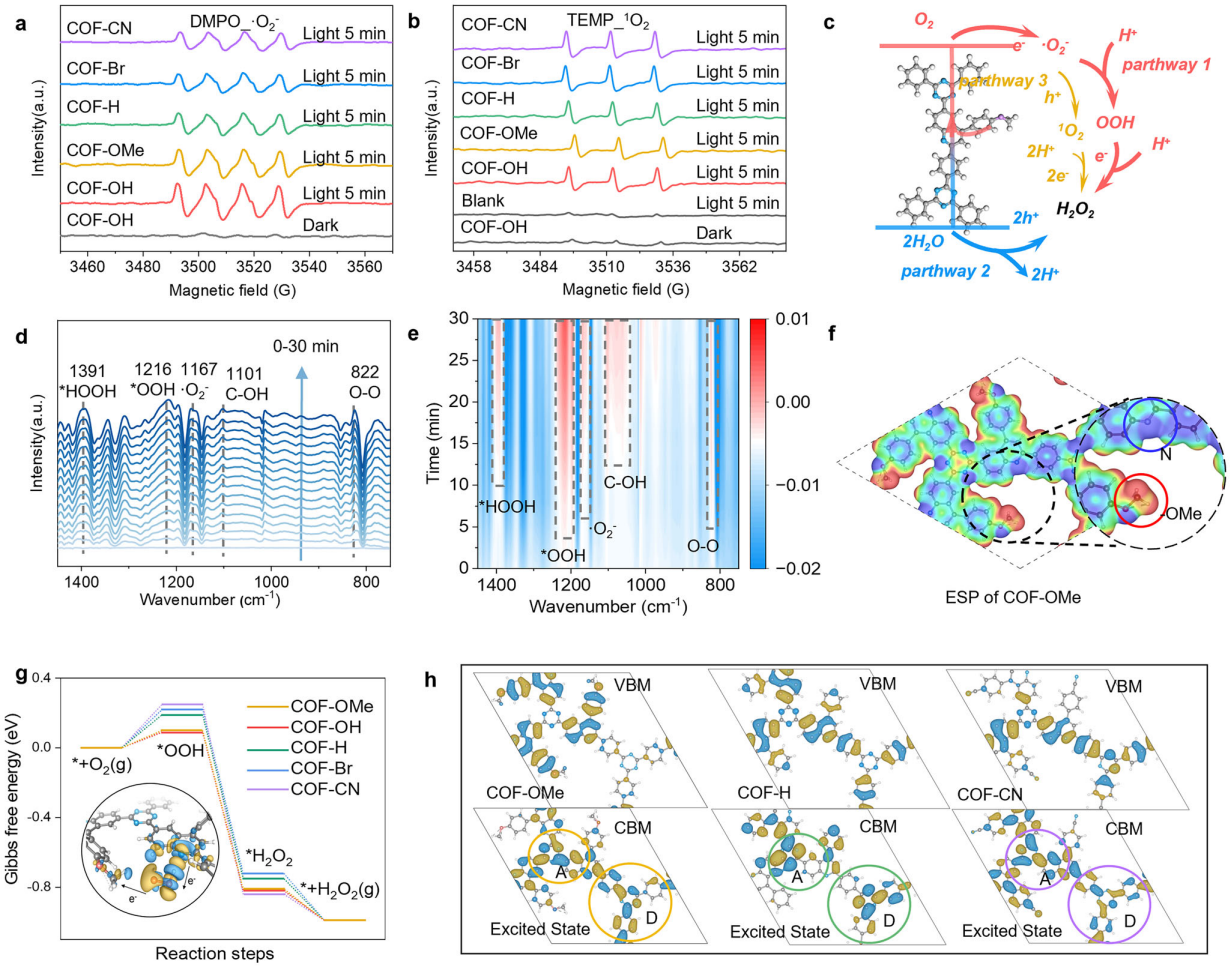
Reaction pathways and mechanisms of H_2_O_2_ photosynthesis. EPR signal of the reaction solution in the dark and under light illumination in the presence of a) DMPO and b) TEMP as the spin‐trapping reagent. c) The proposed mechanism of photocatalytic H_2_O_2_ production on COFs‐R. d) In situ DRIFTs spectrum and e) Infrared contour map for COF‐OMe in H_2_O_2_ photosynthesis. f) Electrostatic potential distribution of COF‐OMe. g) Gibbs free energy diagram of photocatalytic ORR pathways for COFs‐R. h) Calculation of CBM/VBM for the structural segments of COF‐OMe, COF‐H, and COF‐CN.

To investigate the ability of different COFs to generate reactive oxygen species and free radicals under photocatalytic conditions, electron paramagnetic resonance (EPR) spectroscopy was performed. The •O_2_^‐^ and ^1^O_2_ were quantified by 5,5‐dimethyl‐pyrroline‐N‐oxide (DMPO) and 2,2,6,6‐tetramethyl‐4‐piper‐idone (TEMP) as captured, as shown in **Figure** **7**a,b, where the COF‐OH and COF‐OMe have a stronger ability to activate O_2_ to •O_2_^‐^. All COFs‐R show a similar ability to generate ^1^O_2_, indicating the electron‐donating group enhances the performance through more •O_2_^‐^ generation. Furthermore, there was no signal for •OH can be detected, indicating that H_2_O_2_ is mainly produced via a 2e^−^ two‐step routine mediated by •O_2_^‐^ (Figure S14, Supporting Information). To gain a more direct insight into the photoexcited electron behavior of the COFs, we further performed in situ EPR measurements under visible light irradiation to probe the concentration of photoexcited CB electrons (Figure S15, Supporting Information). COF‐OMe exhibited a significantly greater increase in EPR signal intensity (44.4%) compared to COF‐CN (29.7%), indicating more efficient photoinduced charge separation in COF‐OMe.

The capture experiments of the active species were carried out to investigate the influence of different reactive species on H₂O₂ formation. When •OH scavenger (tert‐butanol, TBA) was added, the H_2_O_2_ production remained almost unchanged. The addition of the ^1^O_2_ scavenger (β‐carotene) resulted in a slight decrease in performance. While the yield of H_2_O_2_ was significantly inhibited when the •O_2_^‐^ scavenger (benzoquinone, BQ) and e^‐^ scavenger (AgNO_3_) were added (Figure S16, Supporting Information). These results confirm that •O₂⁻ plays a central role in the formation of H₂O₂, while ¹O₂ indirectly influences H₂O₂ production through pathway 3 (Figure 7c).^[^ ^47^, ^48^ ^]^ The intensities of the characteristic peaks of •O_2_^−^ (1167 cm^−1^) and O−O (822 cm^−1^) increased progressively during the reaction in the in situ diffuse reflectance infrared Fourier transform (DRIFT) spectroscopy tests for COF‐OMe (Figure 7d,e), signifying the adsorption of O_2_ and the occurrence of the two‐step single‐electron pathway in the photocatalytic system based on COF‐OMe. Similar results were observed in COF‐OH, COF‐H, COF‐Br, and COF‐CN, which further indicated that •O_2_^‐^ were key intermediates in the process of photocatalytic generation of H_2_O_2_ (Figures S17–S20, Supporting Information).

To gain a deeper understanding of the catalytic mechanism of COFs‐R, a series of models was constructed for DFT calculations (Figure S21, Supporting Information). Electrostatic potential analysis shows that the ‐OMe substituent donates electrons to the COF backbone, making COF‐OMe exhibit the strongest nucleophilicity, which is favorable for electron donation to O_2_ (Figure 7f; Figure S22, Supporting Information). The adsorption structures of the key polar intermediate *OOH were constructed between the adjacent nucleophilic N sites and ‐R sites in COFs‐R, where the COF‐OMe and COF‐OH exhibit much stronger adsorption energies than the others (Figures S23 and S24, Supporting Information). The charge density difference of COF‐OMe ^*^OOH analysis shows that the polar ^*^OOH gains electrons at the N site and is stabilized between the ‐OMe and ‐N sites. Furthermore, the calculated optimal ORR pathway for COFs‐R shows that the rate‐determining step (RDS) of the entire reaction is the formation of OOH (Figure 7g). Due to the stable adsorption structure of OOH, COF‐OMe, and COF‐OH exhibit the lowest RDS values of 0.10 and 0.09 eV, compared to COF‐Br (0.22 eV), COF‐CN (0.25 eV), and COF‐H (0.19 eV). Notably, the CB minimum (CBM) of COF‐OMe and COF‐OH is localized on the styrene substituents. Upon light excitation, electrons transfer to the VB maximum (VBM) located on the COF backbone, resulting in significant charge separation between the CBM and VBM (Figure 7h; Figure S25, Supporting Information). This reduces the recombination of photogenerated electrons and holes, leading to enhanced photocatalytic performance.

**Figure 8:**
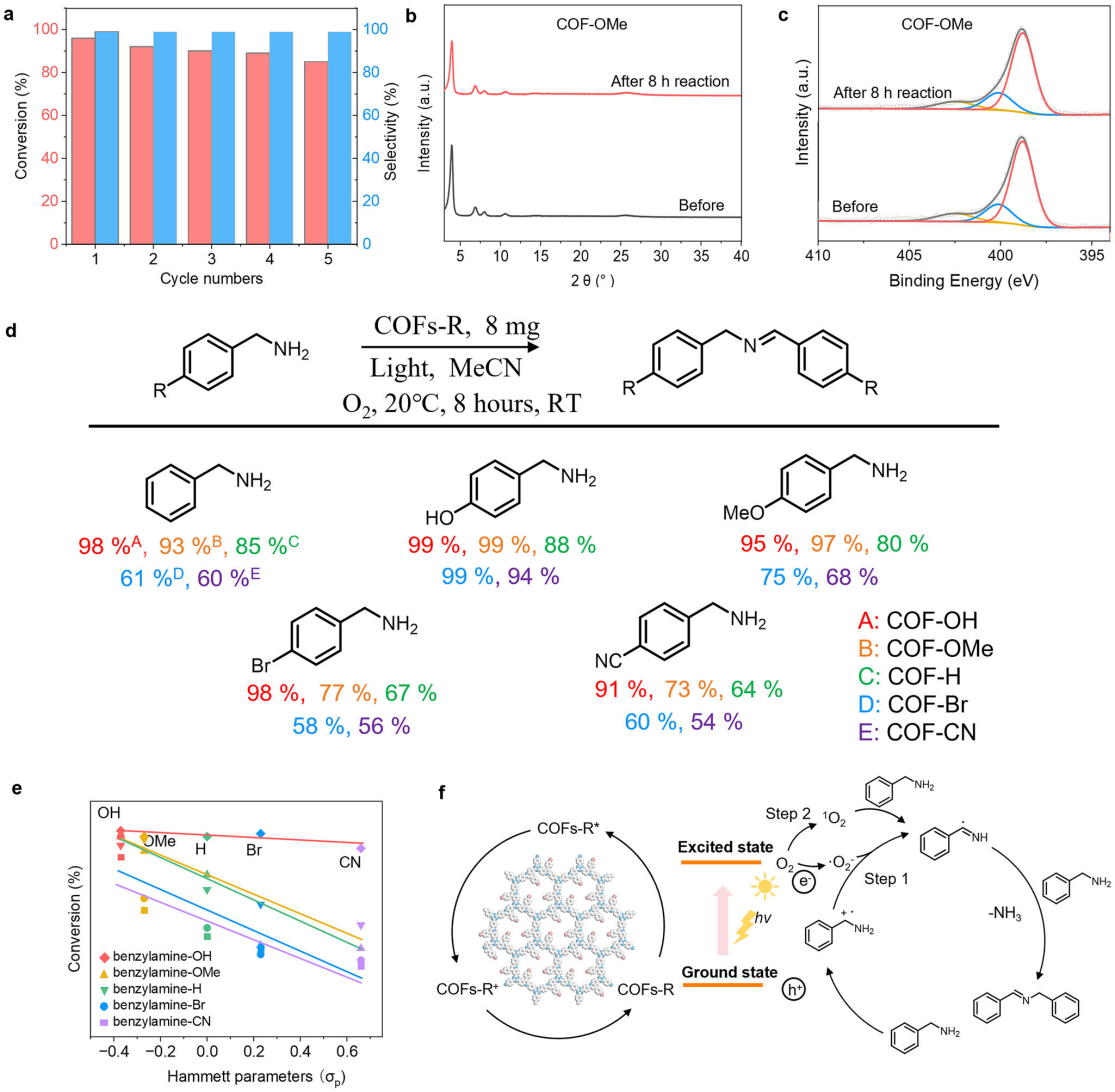
Benzylamine coupling reactions. a) Five runs of benzylamine coupling with COF‐OMe as the photocatalyst. b) PXRD patterns, c) XPS spectra for COF‐OMe before and after photocatalytic reactions. d) Photocatalytic conversion of benzylamine derivatives to N‐benzylidene benzylamine derivatives by COFs‐R. e) The relationship between the *σ_p_* of the substituents in COFs‐R and their performance. f Proposed coupling mechanism of benzylamine.

The photocatalytic oxidation produces valuable products for the chemical and pharmaceutical industry. Recently, various heterogeneous catalysts have been used for such photocatalytic selective oxidations; however, stability and, thus, reusability are critical issues in the presence of highly reactive oxygen species (ROS). Taking COF‐OMe as a photocatalyst, recycling photocatalytic reactions for the coupling reaction of benzylamine was carried out, and the results revealed that no obvious decrease was detected in conversion rates after five cycles of reactions (**Figure** **8**a; Figure S26, Supporting Information). PXRD and XPS measurement was applied to the recycled sample of COF‐OMe, showing that the structure of COF‐OMe was still maintained after the five runs of catalytic reactions (Figure 8b,c). The results not only indicated the high stability of the quinoline‐linked COF‐OMe but also confirmed that the photoactive COF can be used as an effective recyclable photocatalyst in organic catalytic conditions.

The oxidation of different benzylamine derivatives using COFs‐R was further tested; the conversion of benzylamine, 4‐hydroxybenzylamine, and 4‐methoxy benzylamine can reach higher than 90% for COF‐OH and COF‐OMe after 8 h reaction, while the conversion for COF‐H, COF‐Br, and COF‐CN is much lower (Figure 8d; Figures S27–S31, Supporting Information). This trend in the benzylamine coupling reaction shares a similar relationship to the *σ_p_* as the H_2_O_2_ production (Figure 8e). The possible mechanism of coupling reactions was investigated by adding the consuming reagents into the reaction system (Figure S32, Supporting Information).^[^ ^49^ ^]^ The reaction mechanism can be concluded as follows: under visible light irradiation, O_2_ reacts with photogenerated electrons to form •O_2_^‐,^ and ^1^O_2_ was generated via energy transfer from COFs‐R; concurrently, the adsorbed benzylamine molecules undergo oxidation by photogenerated holes, resulting in the formation of benzylamine radical cations. The •O_2_^‐^ species then abstract protons from these benzylamine radical cations, yielding Ph‐CH = NH. This intermediate subsequently reacts with another benzylamine molecule, leading to the formation of benzalaniline as the final product, accompanied by the liberation of ammonia. On the other hand, ^1^O_2_ subsequently captures two protons from benzylamine, generating Ph−CH = NH. Similarly, benzylamine is further generated as a final product by the nucleophilic addition reaction of Ph−CH = NH and benzylamine (Figure 8f).^[^ ^50^, ^51^, ^52^ ^]^

## 3 Conclusion

In summary, we rationally designed quinoline‐based covalent organic frameworks with different functional groups around the quinoline sites as stable and high‐performance photocatalysts for H_2_O_2_ production and benzylamine coupling reactions. As the electronic properties of the functional groups transition from electron‐withdrawing to electron‐donating, the modified quinoline outer‐sphere microenvironment activates the photocatalytic performance, resulting in the highest H_2_O_2_ yield and best benzylamine coupling activities for the strongly electron‐donating groups COF‐OMe and COF‐OH. Theoretical calculations reveal that the linker substituents R provide additional donor structures to the COF triazine framework, thereby enhancing its overall donor architecture and promoting the efficiency of photogenerated carrier separation. Furthermore, electron‐donating groups increase the nucleophilicity of the quinoline sites, facilitating electron transfer to O_2_ and providing optimal binding sites for the key polar intermediate OOH. Furthermore, besides the similar crystallinity and pore structure of all the COFs‐R, the surface area and surface wettability also play important roles in photocatalytic performance. Even the COF‐OH shows the lowest surface area but still shows the highest performance, indicating that the electronic effect of substituents, quantified by Hammett parameters, plays the predominant role in determining the overall photocatalytic performance. More importantly, we have found that such an effect can be quantitatively assessed by the magnitude of Hammett parameters. This will build a feasible bridge for subsequent research into the relationship between the structure and performance of photocatalytic COFs, thereby providing theoretical guidance and an experimental basis for the design and optimization of new photocatalytic materials.

## Conflict of Interest

The authors declare no conflict of interest.

## Supporting information



Supporting Information

## Data Availability

The data that support the findings of this study are available from the corresponding author upon reasonable request.
