# Supplementary material for: Micro‐Environment Programmable Quinoline COFs for High‐Performance Photocatalytic H2O2 Generation and Benzylamine Coupling
Source: Adv Sci (Weinh). 2025 Jul 17;12(33):e05794. doi: 10.1002/advs.202505794 (PMC12412608; doi:10.1002/advs.202505794)
Supplement: Supplementary file 1 — Supporting Information [file ADVS-12-e05794-s001.docx]

Supplementary Materials for

**Micro-environment Programmable Quinoline COFs for High-performance** **Photocatalytic H_2_O_2_ Generation and Benzylamine Coupling**

**Experimental Section**

1. **Materials and Chemicals**

1,3,5-Trimethylbenzene (Mesitylene, 98%), 1,4-Dioxane (Dioxane, 99%), o-DCB (1,2-Dichlorobenzene, 99%) anhydrous n-BuOH (n-Butanol, 99%), 4-Vinylphenol (10% w/w in propylene glycol), 4-Methoxystyrene (95%), styrene (99%), 4-Bromostyrene (95%), 4-Cyanostyrene (97%), 2,3-Dichloro-5,6-dicyano-1,4-benzoquinone (DDQ), boron trifluoride diethyl etherate (BF_3_.Et_2_O), ethanol (EtOH), acetate (6M), 3, 3′, 5, 5′-tetramethylbenzidine (TMB, 99.9%), 5,5-dimethyl-1-pyrroline-N-oxide (DMPO, 97.0%), 2,2,6,6-tetramethylpiperidinooxy (TEMPO), benzoquinone (BQ) was purchased from Aladdin reagents (Shanghai, China). 4,4',4''-(1,3,5-Triazine-2,4,6-triyl)trianiline (TTT, >96.0%), and 2,4,6-Tris(4-formylphenyl)-1,3,5-triazine (TTA, 98.0%) were all supplied by Yanshen Technology Chinese. All other chemicals were obtained from Aladdin reagents and used as received without further purification, and deionized pure water (18.2 MΩ·cm) used in the experiments was produced from a Milli-Q Academic system (Millipore Corp., Billerica, MA, USA).

1. **Synthesis of COF-R**

The synthesis of COF-R is based on the reported literature with modifications.^[1]^ Typically, A Pyrex glass tube (15 mL) was charged with TAB (55 mg, 0.15 mmol), TTA (60 mg, 0.15 mmol), styrene (48 μL, 0.5 mmol), DDQ (5 mg), BF_3_.Et_2_O (8 ul), o-DCB/n-BuOH (1.5/1.5 mL) and 0.2 mL 6 M acetic acid aqueous solution. The tube was first sonicated for 20 minutes to form bulk solid and then flash frozen at 77 K (liquid N_2_ bath) and degassed by three times of freeze-pump-thaw cycles. The internal pressure was evacuated to 10-3 mbar. The tube was sealed and heated at 120 °C for 3 days. Subsequently, COF-OH, COF-OMe, COF-Br, and COF-CN were synthesized by applying the same protocols except for the addition of 4-Vinylphenol (0.58 mL, 0.5 mmol), 4-Methoxystyrene (46 μL, 0.5mmol), 4-Bromostyrene (69 μL, 0.5mmol), 4-Cyanostyrene (62 μL, 0.5mmol), respectively. The precipitate was washed with mixture of solvents (MeOH, acetone) several times and collected after Soxhlet extraction for 12 h. Finally, the powder was vacuum-dried at 80 °C for 8 h.

1. **Synthesis of Im-COF**

A Pyrex glass tube (15 mL) was charged with TTT (55 mg, 0.15 mmol), TTA (60 mg, 0.15 mmol), o-DCB/n-BuOH (1.5/1.5 mL) and 0.2 mL 6 M acetic acid aqueous solution. The tube was first sonicated for 20 minutes and then flash frozen at 77 K (liquid N_2_ bath) and degassed by three times of freeze-pump thaw cycles. The internal pressure was evacuated to 10-3 mbar. The tube was sealed and placed in a preheated oven at 120 °C for 3 days. After finishing heating, the tube was cooled down and cut. The formed yellow precipitate was filtered and washed with acetone/MeOH several times. Finally, the powder was vacuum-dried at 80 °C for 8 h. Yield = 92 % (105 mg).


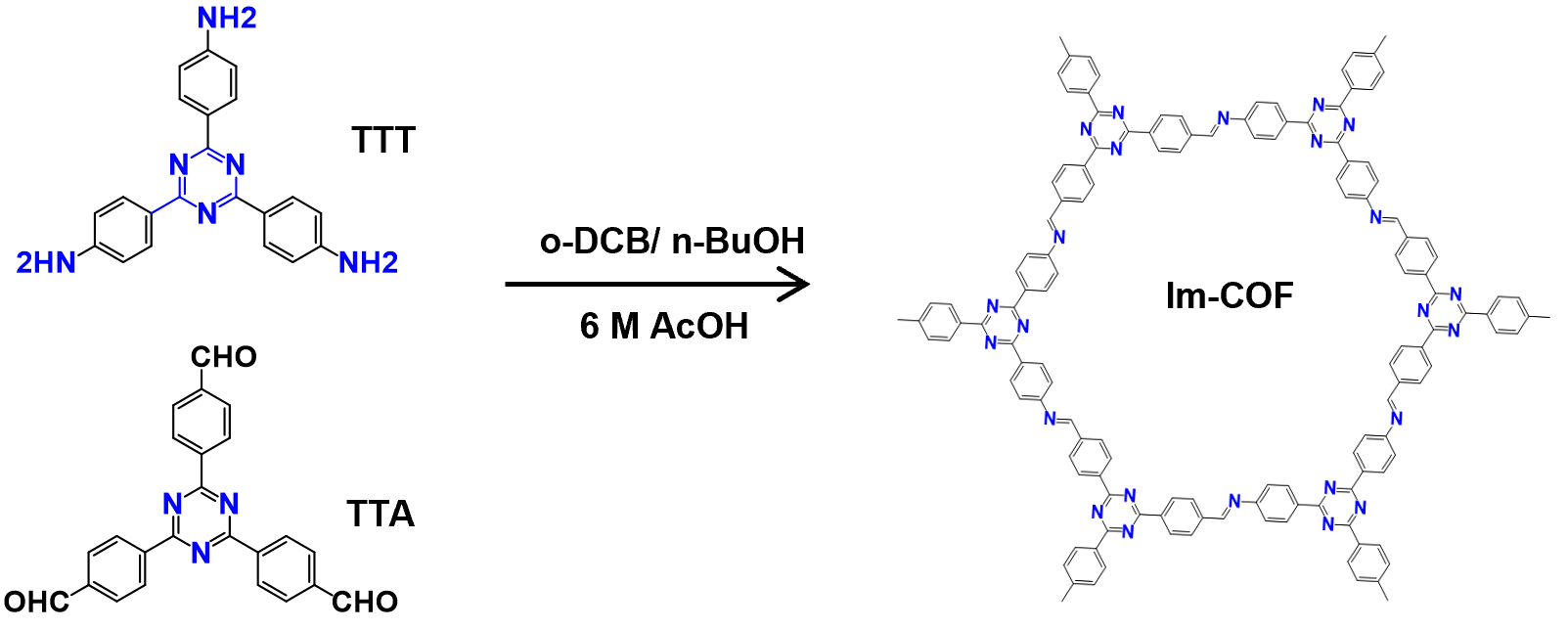


**Scheme S1. Synthesis of Im-COF.**

1. **Characterization**

Aberration-corrected high-angle annular X−ray diffraction (XRD, DX-2700BH, HaoYuan Instrument, China) was used to analyze crystal structures of catalysts with Cu Kα radiation for a 2θ range of 2−40°. A Nicolet-ls50 spectrophotometer (Nicol, US) was used for Fourier transform infrared (FT-IR) spectroscopy analysis in the range of 4000-500 cm^-1^ with a resolution of 2 cm^-1^. N2 sorption analysis was conducted on a Quantachrome Autosorb IQ instrument. All powder samples were degassed at 80 °C overnight before actual measurement. The surface area was calculated by using Brunauer-Emmett-Teller (BET) calculations. The pore size distribution (PSD) plot was recorded from the adsorption branch of the isotherm based on the QSDFT model for slit/cylinder pores. Thermogravimetric analyses (TGA) were carried out on a Q500 TGA (TA) analyzer under Ar atmosphere at a heating rate of 10 ℃·min^-1^ within a temperature range of 50-800 °C. Scanning electron microscopy (SEM) was performed with HITACHI Regulus 8220. The transmission electron microscopy (TEM) and energy dispersive spectroscopy (EDS) mapping were performed via a Talos F200x TEM microscope (FEI Ltd., USA) operated at 200 kV and analyzed by GMS-free analysis. X-ray photoelectron spectroscopy (XPS) spectra were measured on the K-Alpha™ + X-ray Photoelectron Spectrometer System (Thermo Scientific) with a Hemispheric 180° dual-focus analyzer with a 128-channel detector. Inductively coupled plasma mass spectrometry. Solid-state diffuse reflectance ultraviolet-visible spectroscopy (UV-vis) spectra have been collected on a Shimadzu UV-3150. The steady-state PL spectra and time-resolved PL decay measurements were performed via FLS-1000. EPR measurements were performed via the Bruker EPR EMX Plus (Bruker Beijing Science and Technology Ltd, USA) at a frequency of 9.8 GHz (microwave power: 1 mW).

1. **Photocatalytic H_2_O_2_ production experiments**

The reactor was charged with COF powder (5 mg) in water (18 mL) without or with a sacrificial reagent (IPA, EtOH, or BA) (2 mL) (water: SA = 18:2), and then ultrasonicated for 10-15 min (to disperse the COF) after being capped under air. For oxygen atmosphere, the flask was continuously purged with O_2_ in each case. The photocatalytic H_2_O_2_ evolution experiments were performed on an Oriel Solar Simulator 300 W Xe lamp (λ≥420 nm, 300 mW/cm^2^). Solution was taken by a syringe equipped with filter at variable time.

1. **The measurement of H2O2**

The production of H_2_O_2_ was measured by iodometry (Scheme. S2).^[2]^ Specifically, 0.2 mL of samples was added to the mixture of 0.5 mL of 0.4 M KI and 0.5 mL 0.1 M potassium hydrogen phthalate (C_8_H_5_KO_4_), which was kept for 30 min.^[3]^ Under acidic conditions, H_2_O_2_ can react with I^–^ to generate triiodide anions (I_3_^–^), which exhibited absorption at 350 nm. Thus, the absorbance at 350 nm by using UV-vis spectroscopy can measure the amount of I_3_^–^, which can further determine the amount of H_2_O_2_.


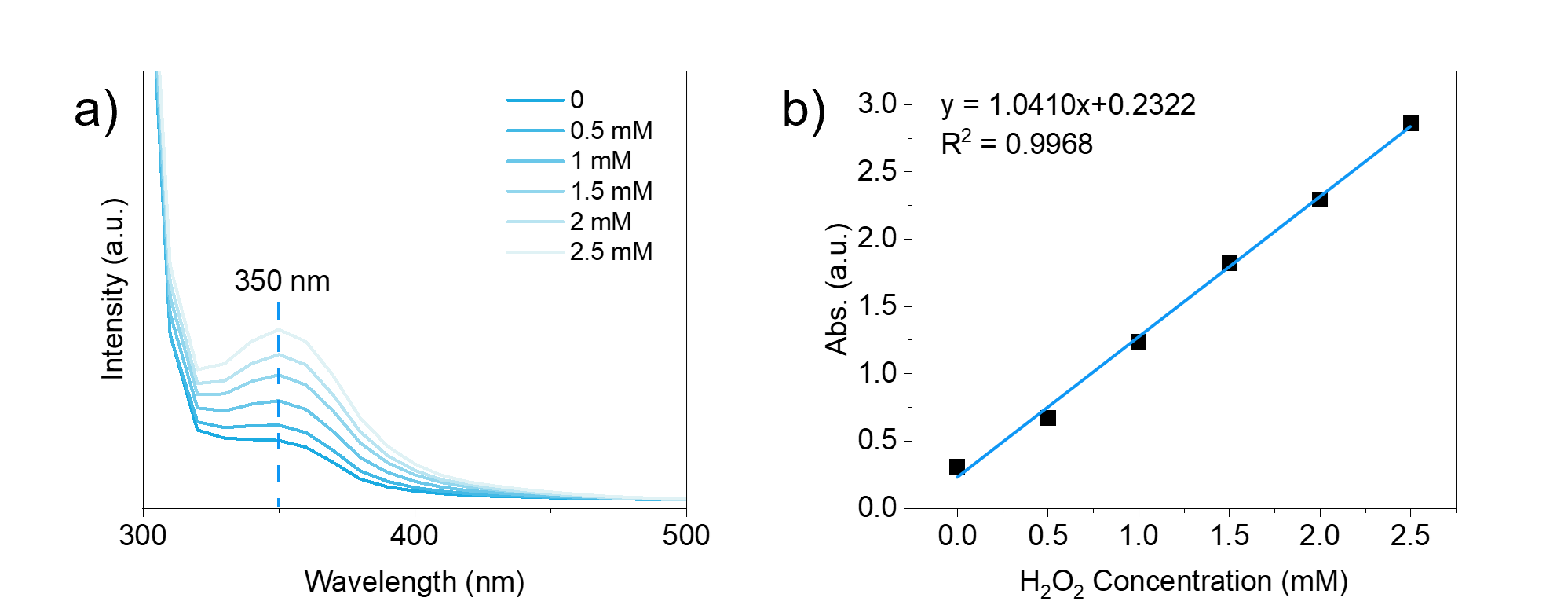


**Scheme S2.** (a) The absorption spectra of H_2_O_2_ at different concentrations (0 µM, 0.5 µM, 1 µM, 1.5 µM, 2 µM, 2.5 µM) at wavelengths of 300-500 nm; (b) and a standard curve of H_2_O_2_ concentration-absorbance was drawn.

1. **AQY Measurements**

The apparent quantum yield (AQY) was determined under LED light irradiation (with band filter) at a certain wavelength (λ = 400 nm, 420 nm, 450 nm, 500 nm, and 675 nm), and the light intensity was measured by a ThorLabs PM100D Power with a photodiode sensor. The AQY was calculated using the following equation:

$$\text{AQY \% =}\frac{\left[ \text{H}_{\text{2}}\text{O}_{\text{2}}\text{ produce }\left( \text{mol} \right) \right]\text{ x}\text{ 2}}{\text{Photon number entered into the reactor }\left( \text{mol} \right)} x 100\% (1)$$

$$=\frac{[N_{a} x h x c] x [H_{2}O_{2} produce (mol)] x 2}{I x S x t x \lambda} x 100\% (2)$$

Where, *N_a_* is Avogadro’s constant (6.022 × 10^23^ mol^-1^), *h* is the Planck constant (6.626× 10^-34^ J s), *c* is the speed of light (3 × 10^8^ m s^-1^), *I* is the intensity of irradiation light (300 W cm^-2^), *S* is the irradiation area (12.56 cm^2^), *t* is the photoreaction time (3600 s), λ is the wavelength of the monochromatic light (m).

1. **SCC Measurements**

According to the experimental method,^[4]^ the solar-to-chemical energy conversion (SCC) efficiency was determined by the photocatalytic experiments using an AM 1.5G spectrum as the light source (300 mW cm^-2^). After air bubbling, the photocatalytic reaction was carried out in pure deionized water (40 mL) with photocatalyst (30 mg). The SCC efficiency was calculated via following equation:

$$SCC\left( \% \right)= \frac{\left[ \Delta G \mathrm{for} H_{2}O_{2} \mathrm{generation} \left( J \mathrm{mol}^{-1} \right) \right] x [H_{2}O_{2} produce (\mathrm{mol})]}{t x S x I_{cut420}} x 100\% (3)$$

wherein, *ΔG* (117 kJ mol^-1^) is represent the free energy of H_2_O_2_ generation; *t* (3600 s) is represent the irradiation time; *S* (12.56 cm^2^) is represent the irradiation area; *I_cut420_* (300 mW cm⁻²) is represent the incident light intensity, respectively.

1. **Electron Paramagnetic Resonance Measurements**

Spin trapping EPR tests were recorded using a Bruker EMX plus spectrometer operating at the X-band frequency (9.8 GHz). 5,5-Dimethyl-1-pyrroline N-oxide (DMPO) was used as a spin-trapping reagent to detect ·OH or ·O_2_^-^. In particular, the catalysts (5 mg) were dispersed into water or a MeOH/water mixture (9/1 v/v, 500 μL) containing DMPO (0.1 mmol) with a Pyrex glass tube, which was sealed with a rubber septum cap. A Xe lamp (λ > 420 nm) was used as the light source. The dispersion was purged with Ar or O_2_ gas for 5 min before light irradiation. 1O2 capture tests were conducted in a methanol-free system, using the same method as described above, but with the use of 2,2,6,6-tetramethylpiperidine (TEMP) as the spin-trapping agent.

1. **Photoelectrochemical measurements**

All the electrochemical measurements were carried out in a conventional three-electrode cell using the Gamry reference 600 workstations (Gamry, USA) at room temperature. Mott Schottky, photocurrent, and Electrochemical Impedance Spectroscopy (EIS) were tested in Na2SO4 solution (0.5 M, pH 7). The working electrode is an Indium-Tin Oxide (ITO) glass plate coated with catalyst slurry; the counter electrode is platinum foil, and saturated Ag/AgCl is the reference electrode. Mott Schottky plots were measured at alternating current (AC) frequencies of 500 Hz, 1000 Hz, and 1500 Hz. Working electrode preparation: 10 mg of catalyst, 1 mL of ethanol, and 10 μL of Nafion were mixed and sonicated for 20 min. 50 μL of slurry was deposited evenly on the ITO glass plate (1 ×1 cm^2^), which was dried under infrared irradiation.

1. **Rotating disk electrode (RDE) measurements**

The electron transfer number for oxygen reduction reaction (ORR) was measured on a rotating disk electrode (RDE) in an O_2_ saturated Na_2_SO_4_ (0.5 mol L^-1^) system with different rotating speeds. The average numbers of electrons (n) were calculated by the Koutecky-Levich equation:^[5]^

$$\frac{1}{i}=\frac{1}{i_{k}}+\left[ \frac{1}{0.620nF{AD}^{2/3}v^{{-1}/6}C} \right]\omega^{{-1}/2} (4)$$

Here, *i* and *i_k_* are the current density (μA cm^-2^) and kinetic current density (μA cm^-2^), respectively; n is the number of electron transfer; *F* is the Faraday constant (96485 C mol^-1^); *A* is the working electrode area (0.196 cm^2^); *D* is the oxygen diffusion coefficient (1.93×10^-5^ cm^2^ s^-1^); *v* is the kinematic viscosity of the electrolyte (0.0109 cm^2^ s^-1^); *C* is the saturated oxygen concentration in water (1.26×10^-3^ M); ω is the rotating speed.

1. **X-ray Powder Diffraction**

Patterns were collected on a Bruker D8 Advance diffractometer in reflection geometry operating with a Cu Kα anode (λ = 1.54178 Å) operating at 40 kV and 40 mA. Samples were ground and mounted as loose powders onto a Si sample holder. PXRD patterns were collected from 2 to 40 2θ degrees with a step size of 0.01 degrees and an exposure time of 1 second per step. The structural crystal models with hcb topology of all the COFs were initially constructed in hexagonal unit cell in the Materials Studio suite of programs by Accelrys. Geometry optimization of the structures with Universal Force Field (UFF) led to satisfactory models whose theoretical pattern matched well the experimentally obtained patterns in terms of reflection positions and relative intensities. The Pawley profile refinements were performed using a Pseudo-Voigt profile function. The observed diffraction patterns were subjected to a polynomial background subtraction and the refined parameters included the zero-point shift, the unit cell parameters, the FWHM parameters and the peak asymmetry (Berar-Baldizzoni function). For all the COFs, AA stacking and AB stacking models were constructed, and their corresponding PXRD patterns were calculated except. We chose to represent the AA structural model of the COFs in the fully eclipsed configuration (AAe).

1. **DFT calculations methods.**

A vacuum layer of 15 Å was simultaneously set along the z-direction of COFs-R. Spin-polarized DFT simulations were conducted within the all-electron, frozen-core projector-augmented-wave (PAW)^[6]^ formalism with periodic boundary conditions and were performed using VASP version 5.4.4. The generalized gradient approximation (GGA) of Perdew−Burke−Ernzerhof (PBE) functional was used.^[7]^ Planewaves, with a kinetic energy cutoff of 520 eV, were used as the basis set. Gaussian electronic smearing with a 0.05 eV width aided electronic convergence. The self-consistent calculations apply a convergence energy threshold of 10^-5^ eV. The maximum Hellmann-Feynman force for each ionic optimization step is 0.02 eV/Å. The Brillouin zone was sampled using 2×2×9 for cell models and 2×2×1 for slab models. We systematically performed a Bader charge analysis after each relaxation using the tools provided in reference.^[8]^

The thermal correction, including zero-point vibrational energy, was carried out by VASPKIT version 1.5.1 for the Gibbs free energy ($\text{∆}\text{G}_{\text{T}}$) of all the adsorbed intermediates and gas molecules.^[9]^

The equations are as the following:

$$\text{E}_{\text{ZPE}}\text{=}\frac{hv}{\text{2}}$$

$$\text{∆G}_{\text{T}}\text{=}\text{E}_{\text{ZPE}}\text{+∆}\text{U}_{\text{0→T}}\text{+TS}$$

The zero-point $\text{∆}$vibrational energy (E_ZPE_) was calculated from the vibrational frequencies $v$ of the adsorbed intermediates. $\text{∆}\text{U}_{\text{0→T}}$ means the correction of internal thermal energy U.

**Supplementary Figures**


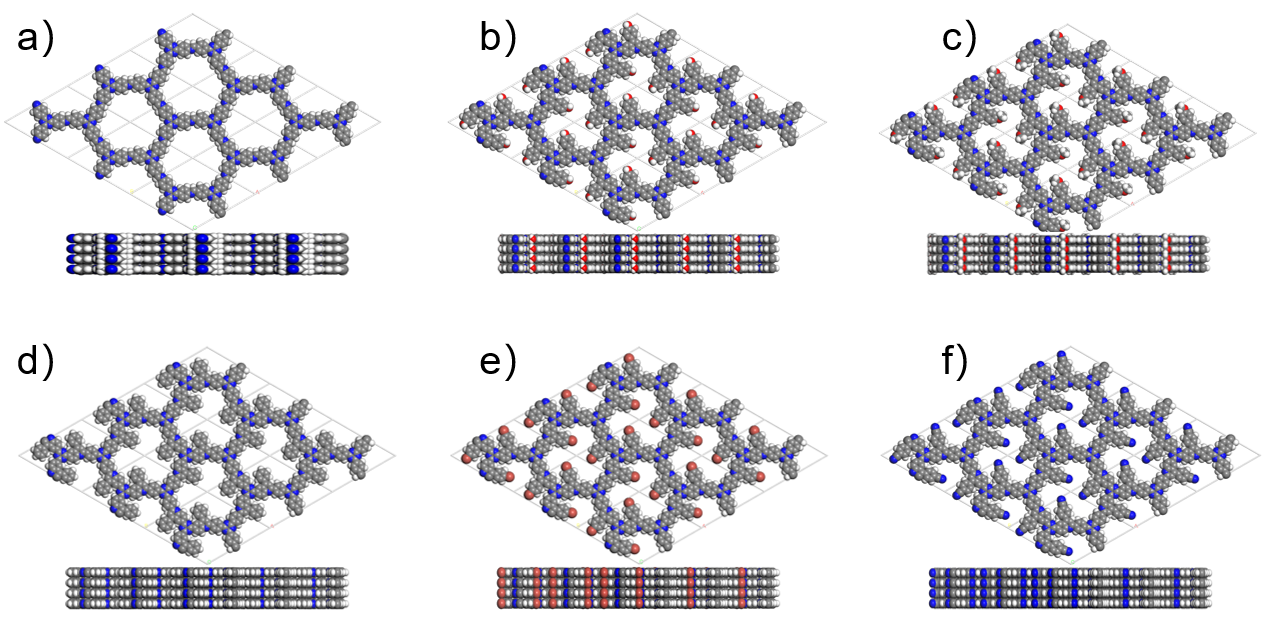


1. Simulated hcb 2D hexagonal layered model with eclipsed (AA) stacking arrangement of Im-COF (a), COF-OH (b), COF-OMe (c), COF-H (d), COF-Br (e) and COF-CN (f).


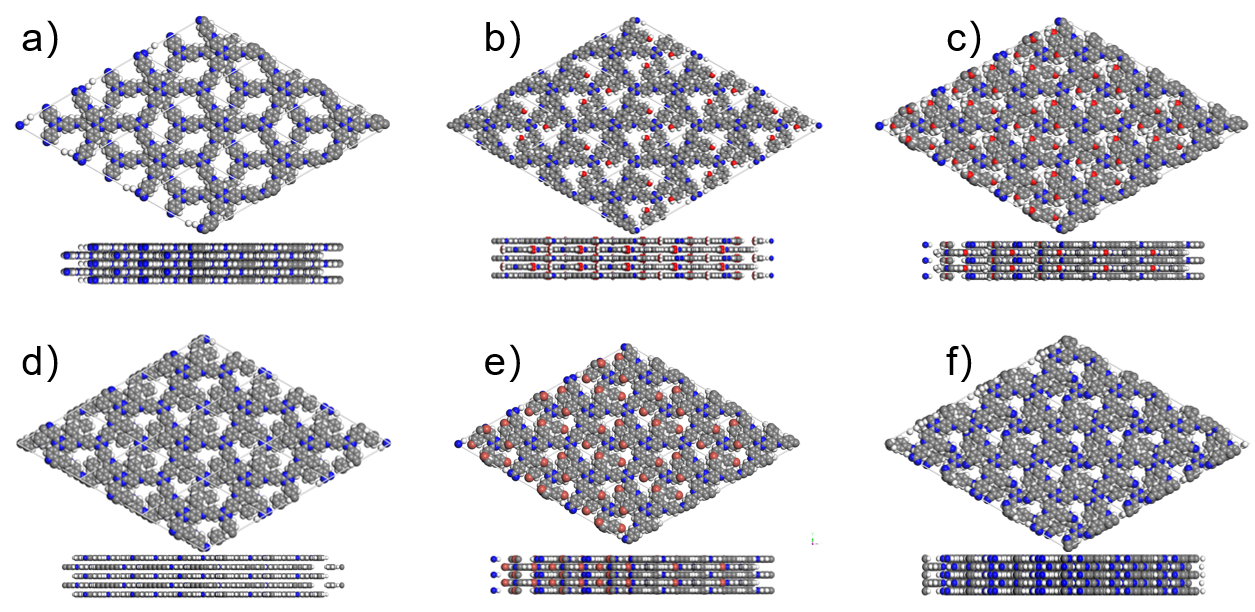


1. Simulated hcb 2D hexagonal layered model with staggered (AB) stacking arrangement of Im-COF (a), COF-OH (b), COF-OMe (c), COF-H (d), COF-Br (e) and COF-CN (f).


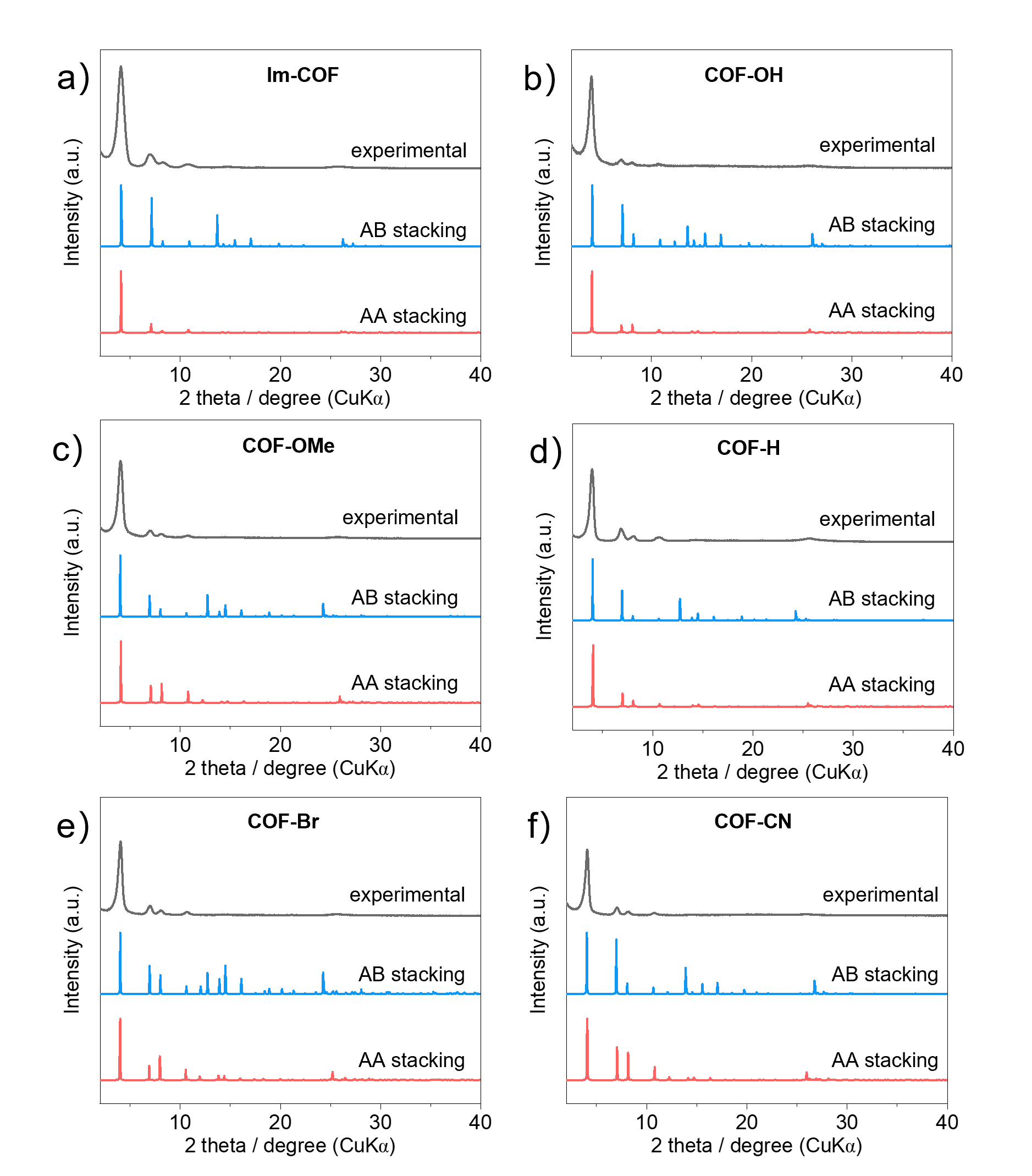


1. Simulated X-ray diffraction patterns for generated hcb hexagonal layered structures adopting fully eclipsed (red) and staggered (blue) stacking arrangement compared to the experimentally obtained patterns (black) of Im-COF (a), COF-OH (b), COF-OMe (c), COF-H (d), COF-Br (e) and COF-CN (f).


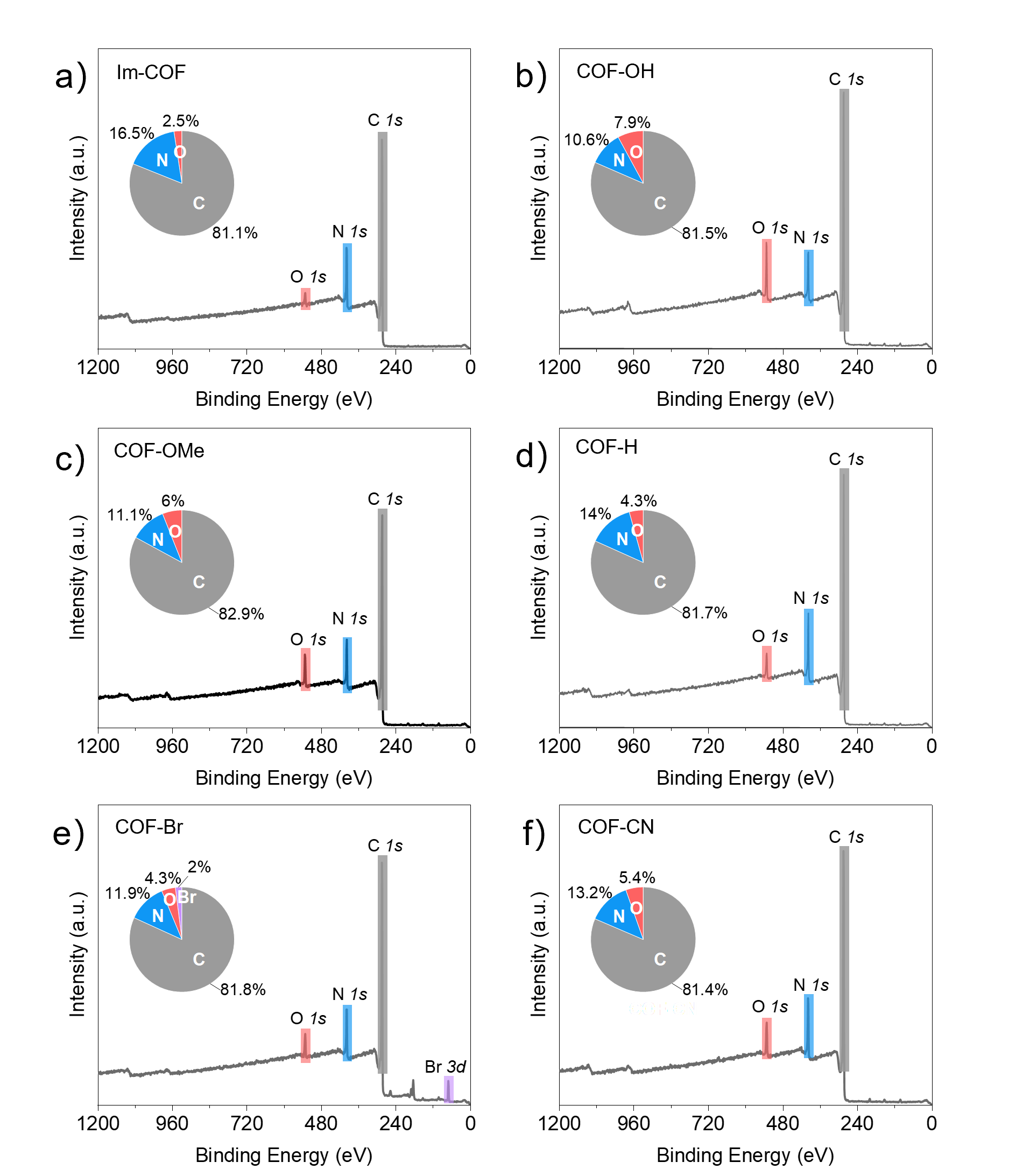


1. XPS survey spectrum of Im-COF (a), COF-OH (b), COF-OMe (c), COF-H (d), COF-Br (e) and COF-CN (f).


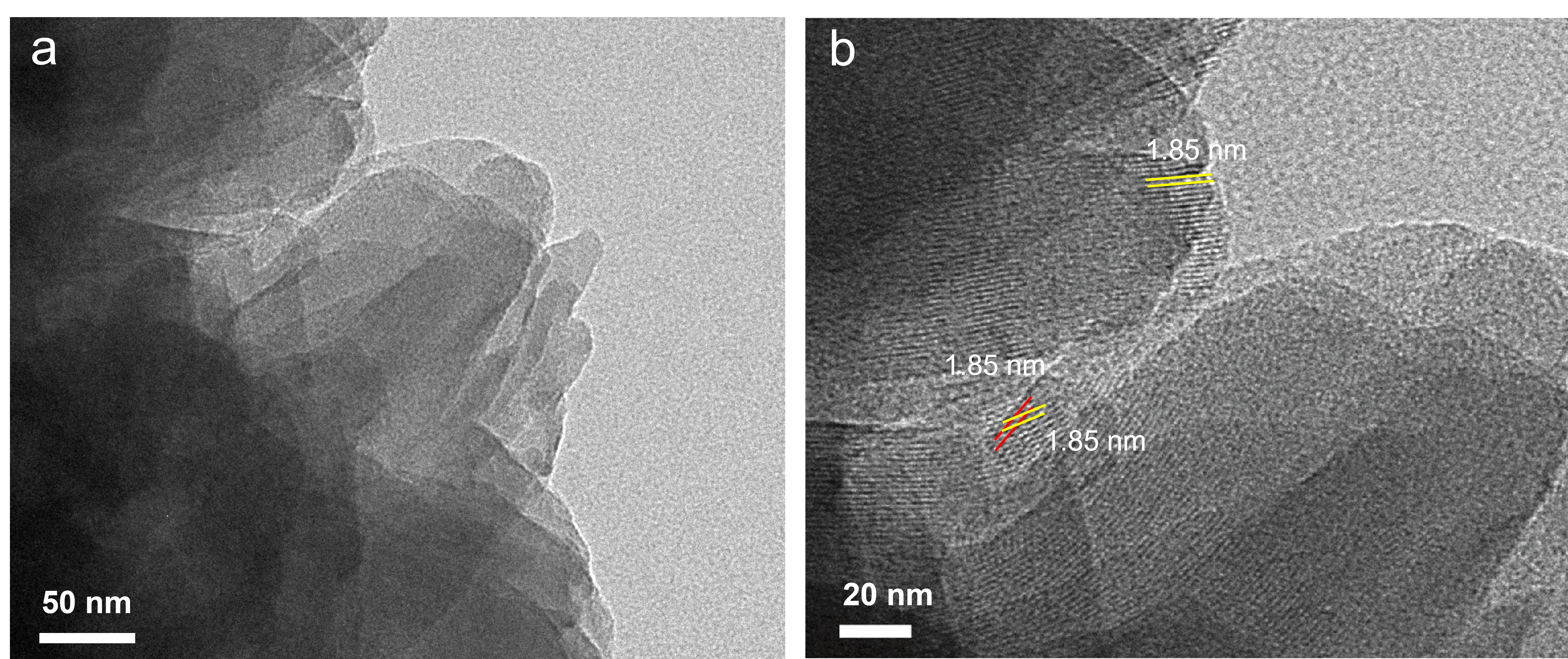


1. a, b) TEM images of quinoline-linked COF-OMe.


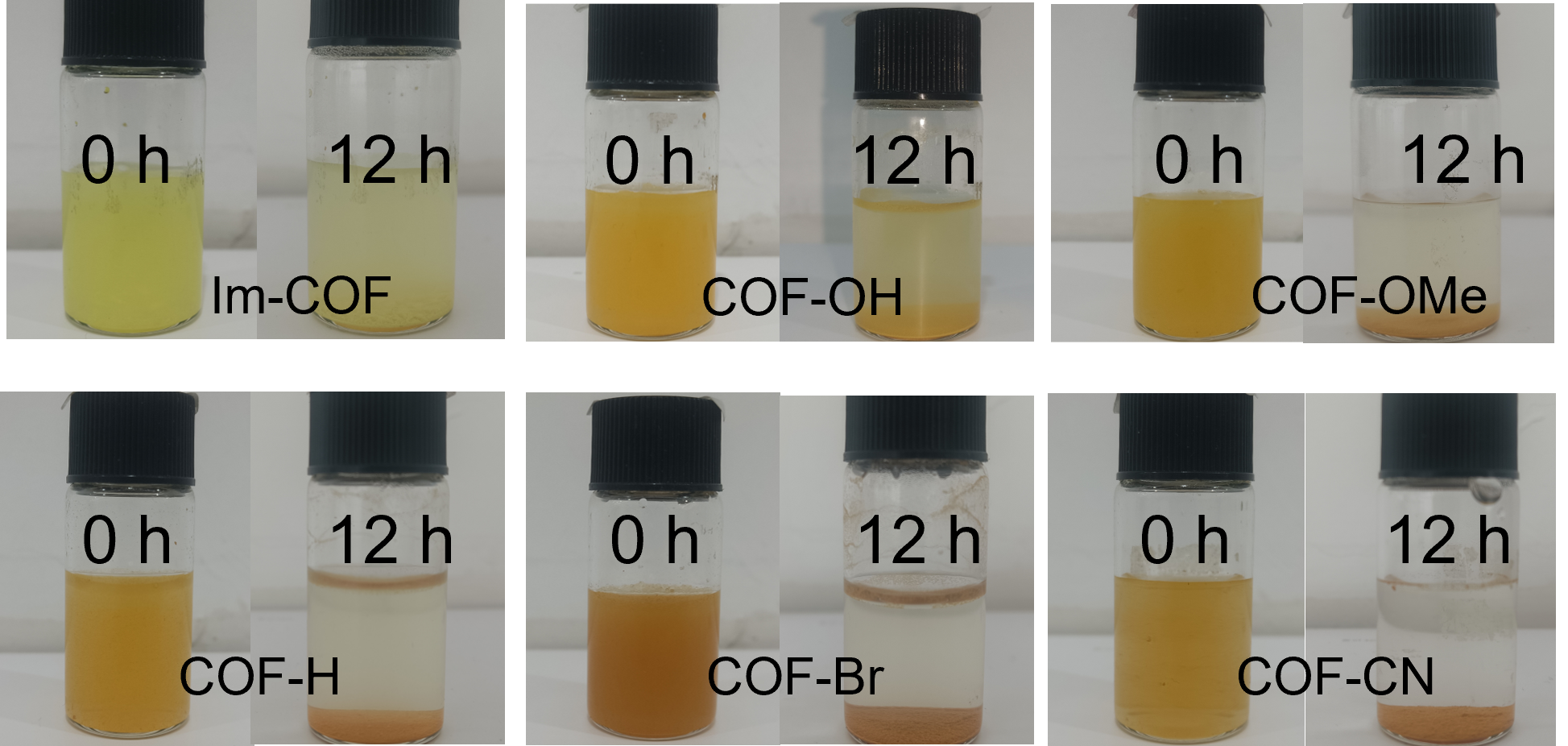


1. Experimental pictures of free sedimentation in ultrapure water (concentration of 1 g/L).


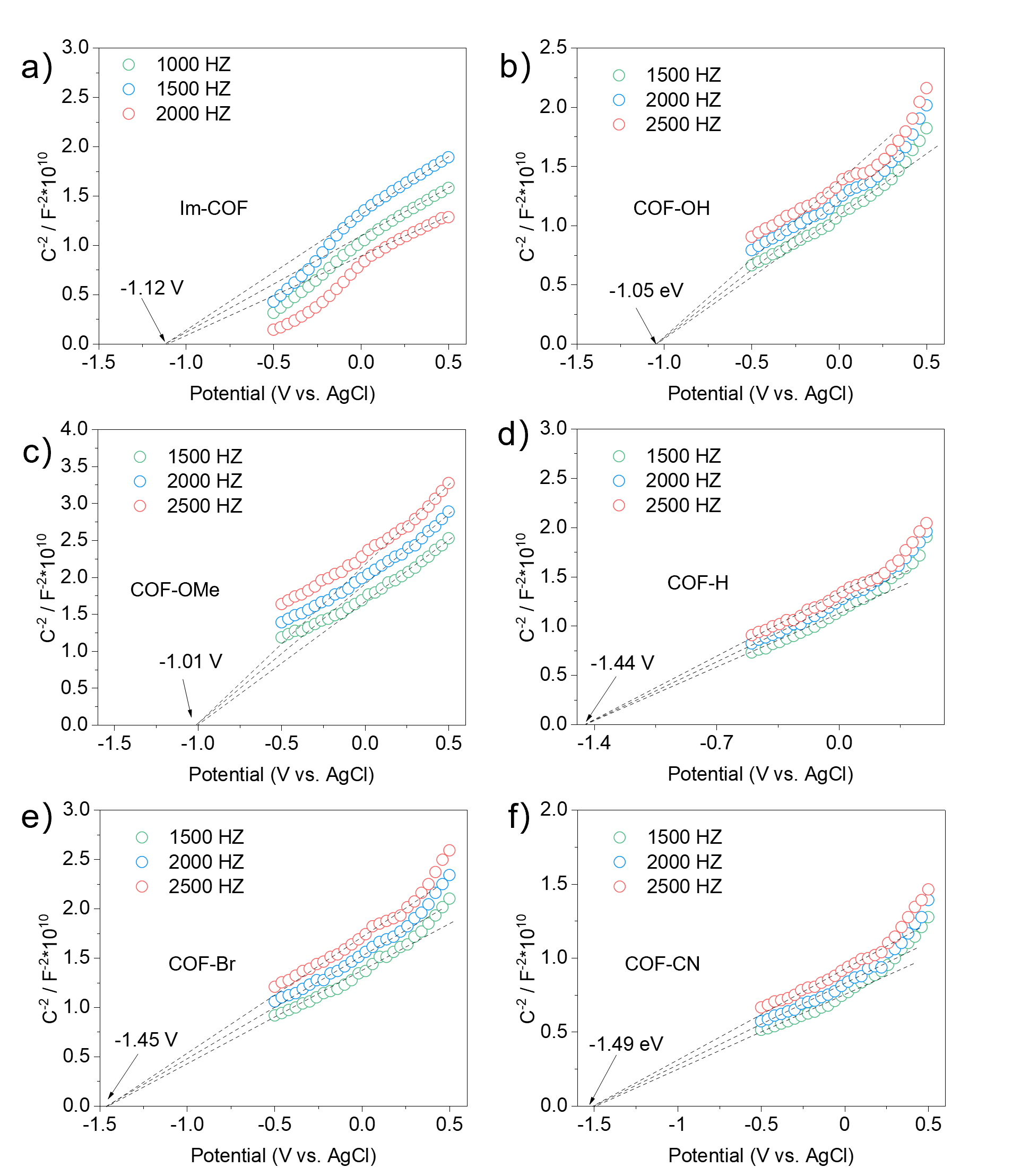


1. Mott-schottky curves of Im-COF (a), COF-OH (b), COF-OMe (c), COF-H (d), COF-Br (e) and COF-CN (f).


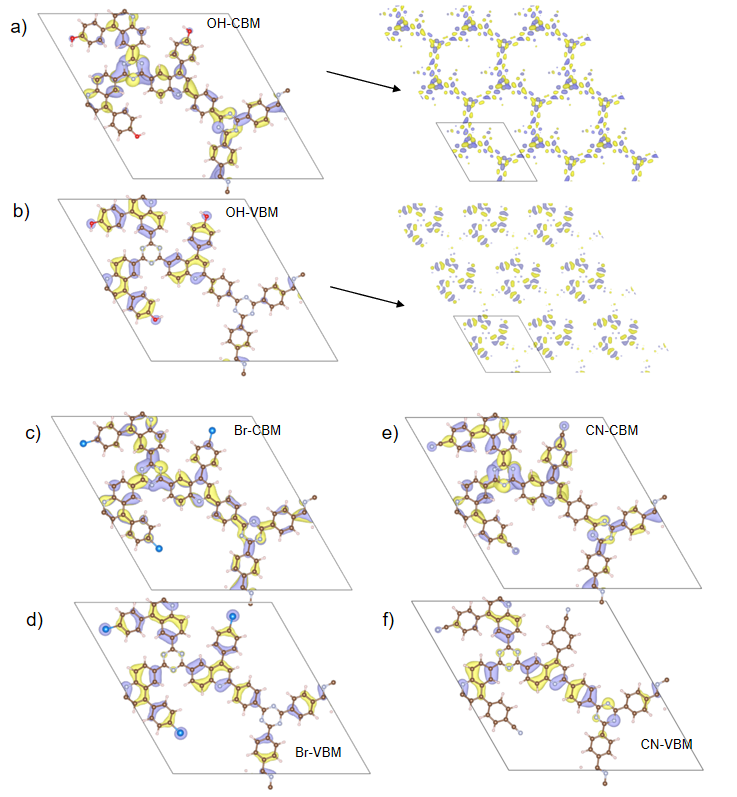


1. HOMO (down), LUMO (up) orbit distribution of COF-OH.


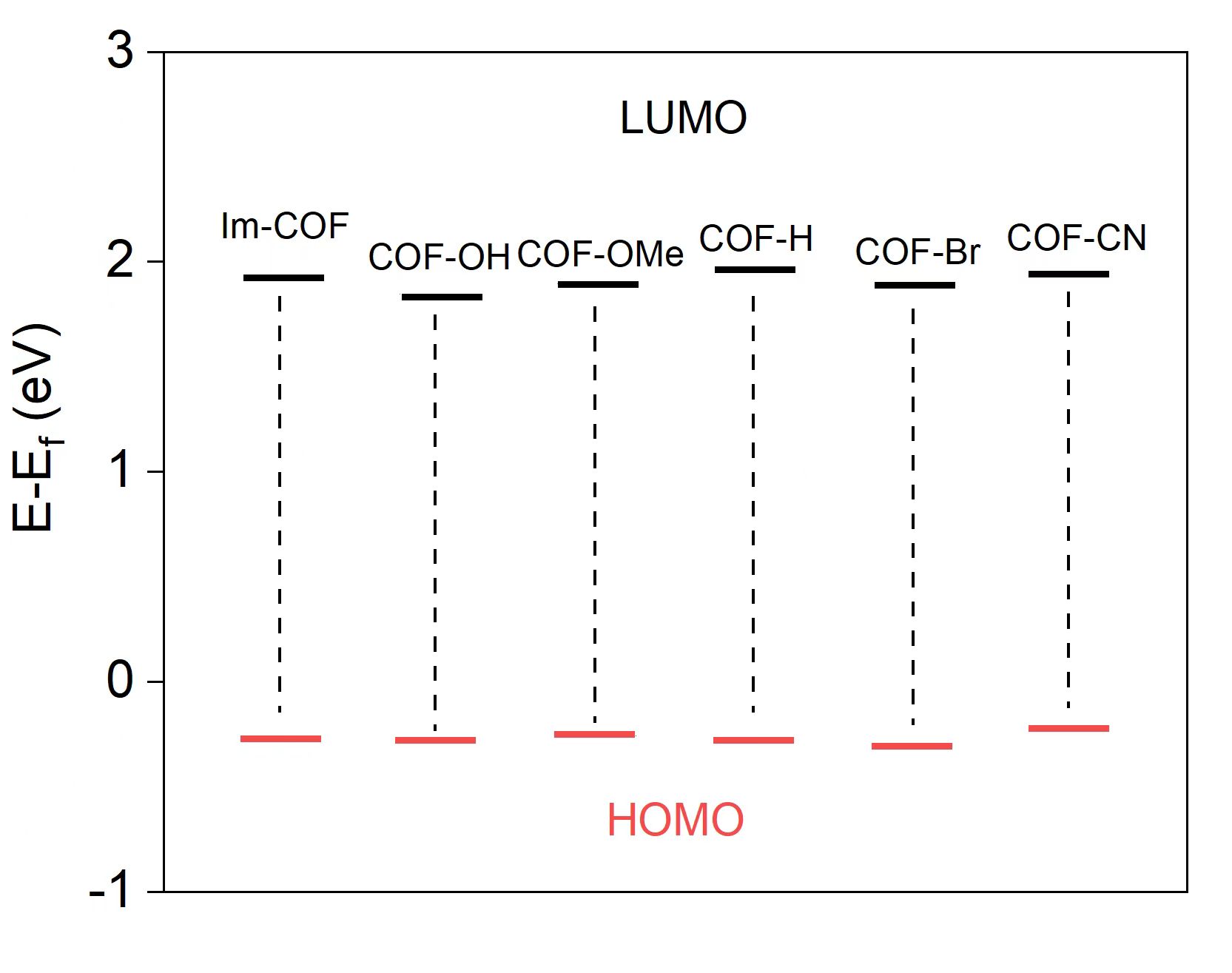


1. Band gaps calculated by DFT for Im-COF and COFs-R.

1. Electrochemical impedance spectroscopy (EIS) Nyquist plots of the samples measured in 0.5 M Na_2_SO_4_ under Xe lamp at a bias of 1.5 V (versus Ag/AgCl).


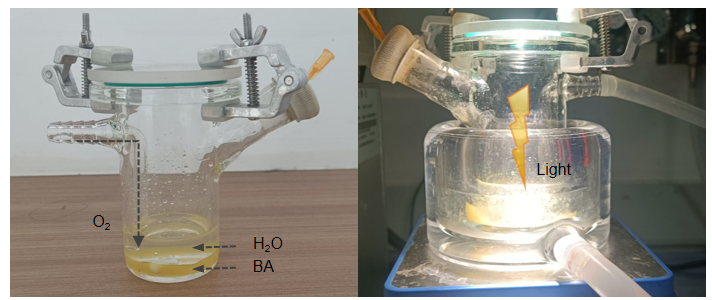


1. Experimental setup for two-phase photo-catalytic system


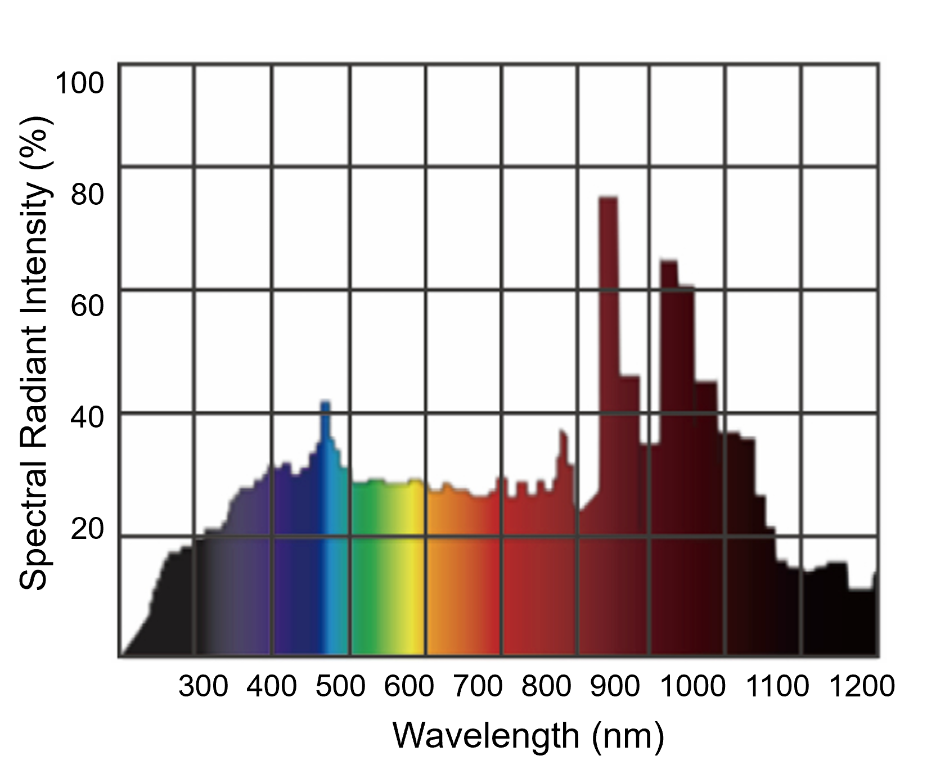


1. Xenon lamp light spectra ozone free lamp used for this experiment. (<https://www.aulight.com/public/ziliao/20201023/>[fbd2d28d9726318a24816402c9d1e31e.pdf](https://www.aulight.com/public/ziliao/20201023/fbd2d28d9726318a24816402c9d1e31e.pdf))

1. Photocatalytic H_2_O_2_ production for Im-COF in water/BA and water.


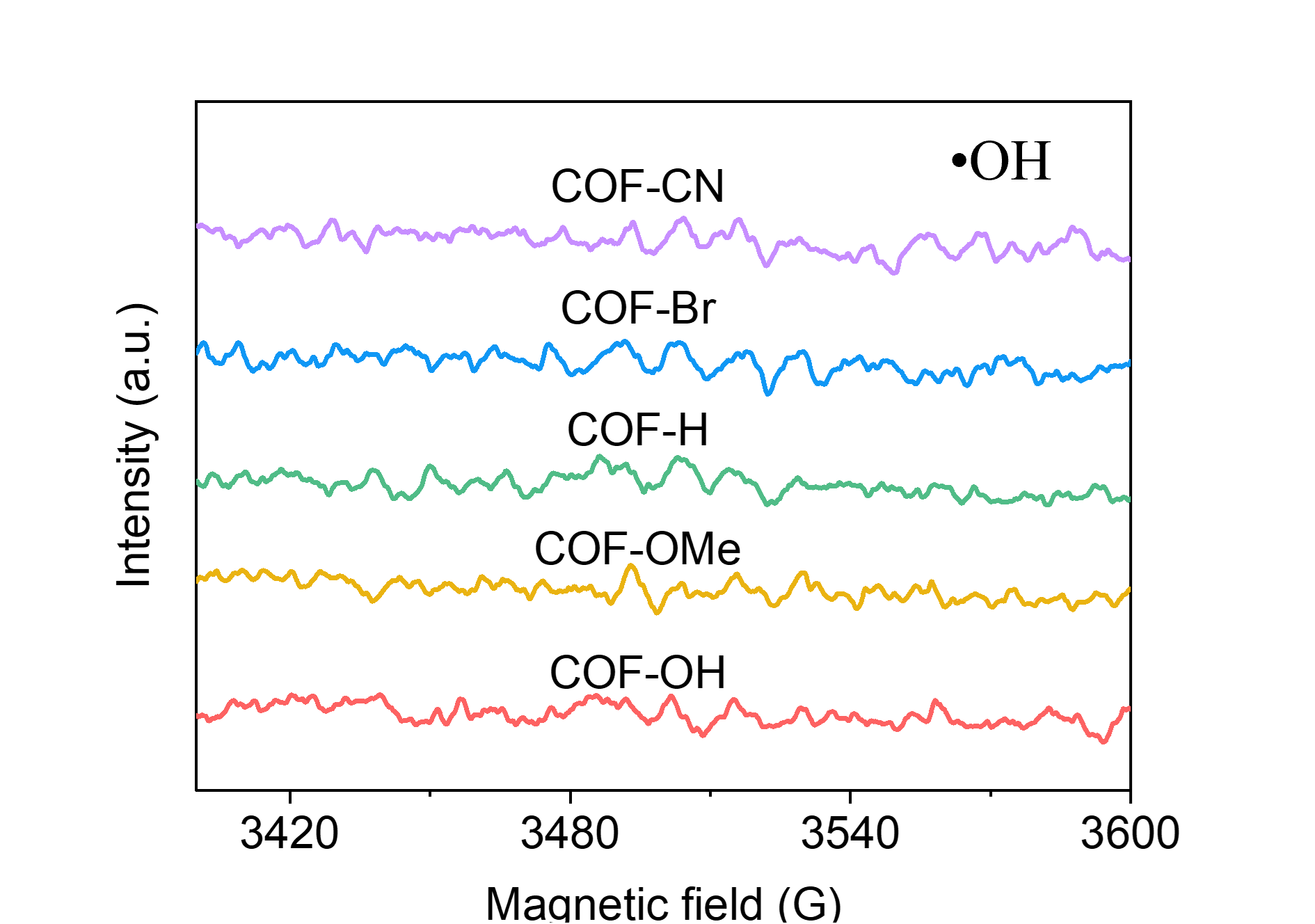


1. DMPO spin trapping EPR spectra. DMPO spin trapping EPR spectra of COFs-R for measuring •OH under visible light (10 min).


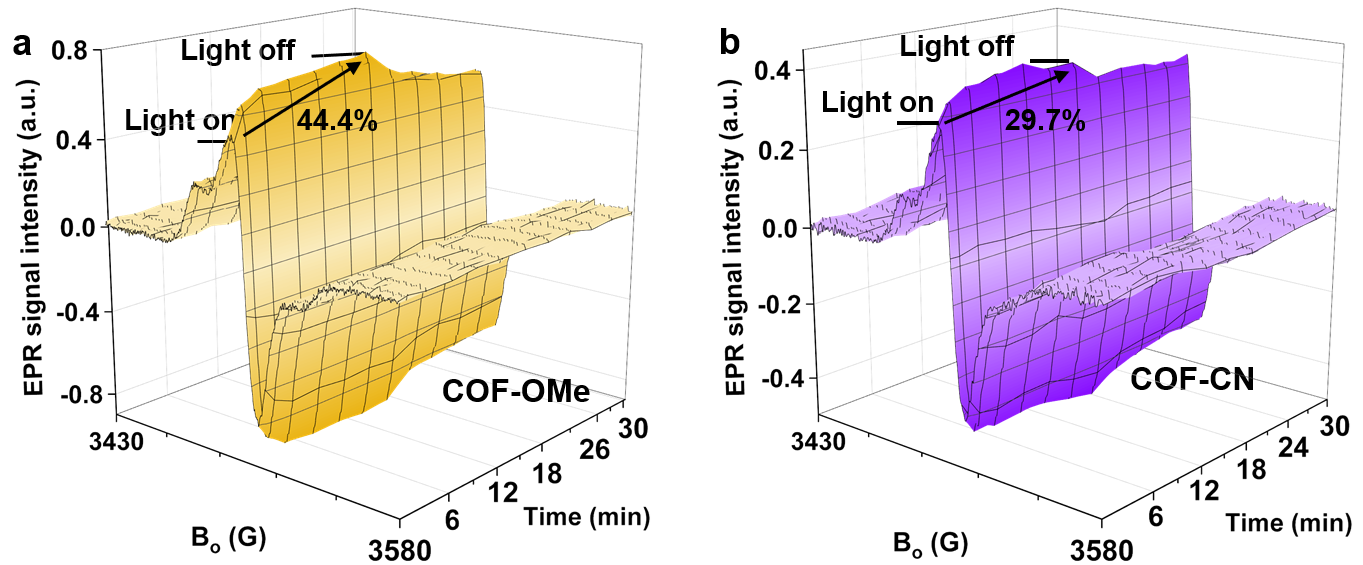


1. EPR conduction band (CB) electrons spectra of COF-OMe **a)** and COF-CN **b)** during and after visible light irradiation.


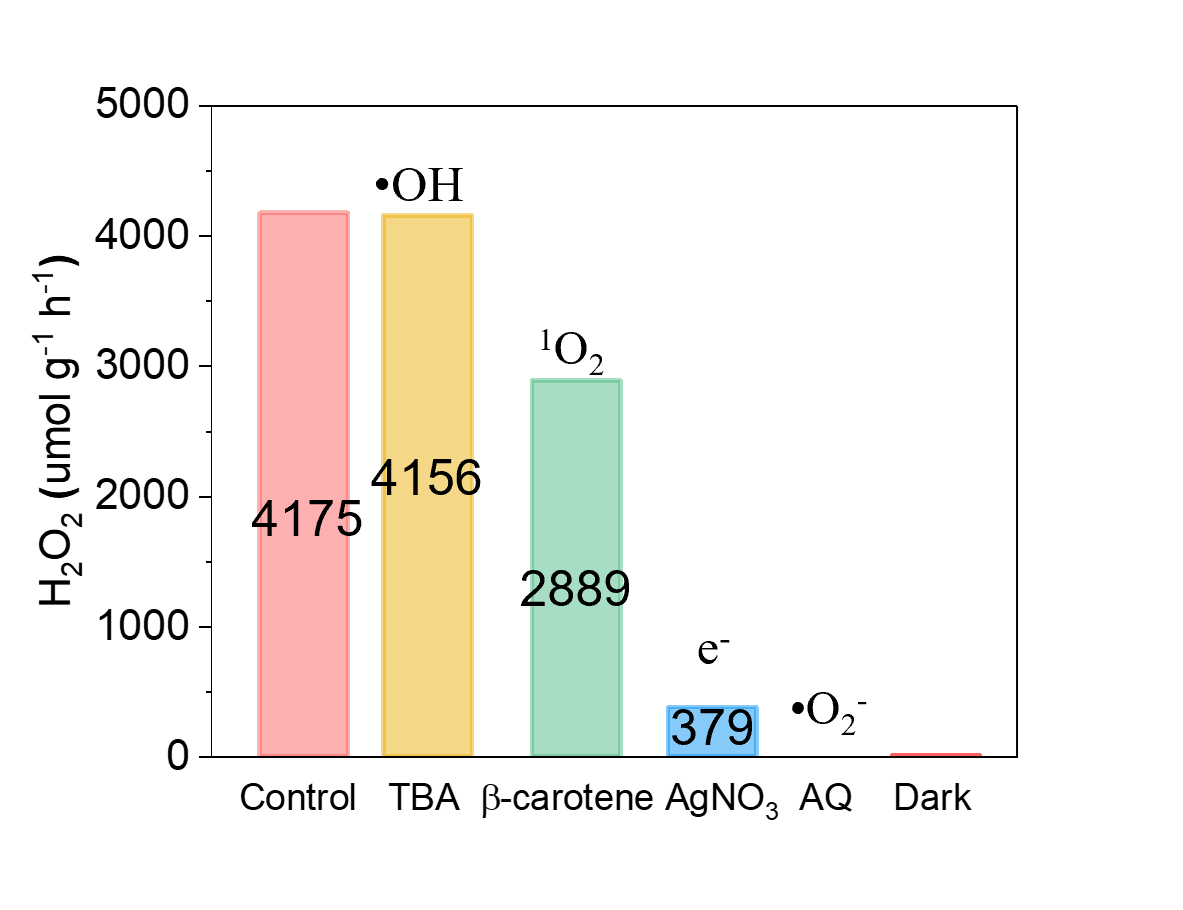


1. Photocatalytic H_2_O_2_ production for COF-OMe in water (Control), water/TBA, water/β-carotene, water/AgNO_3_, and water/BQ, (20 mL, 10 mM aqueous solution, 5 mg COF), all with 1 h illumination (300 W Xe lamp, > 420 nm).


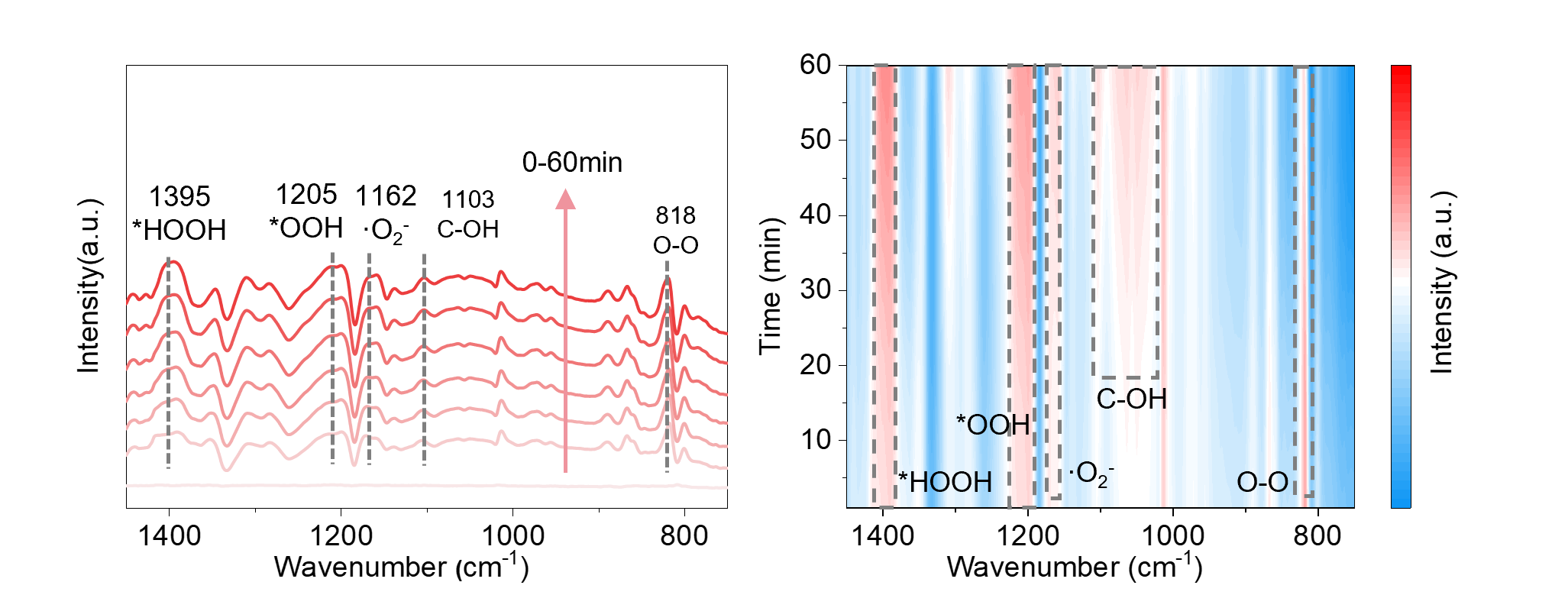


1. In situ DRIFTs spectrum (left) and infrared contour map (right) for COF-OH in H_2_O_2_ photosynthesis.


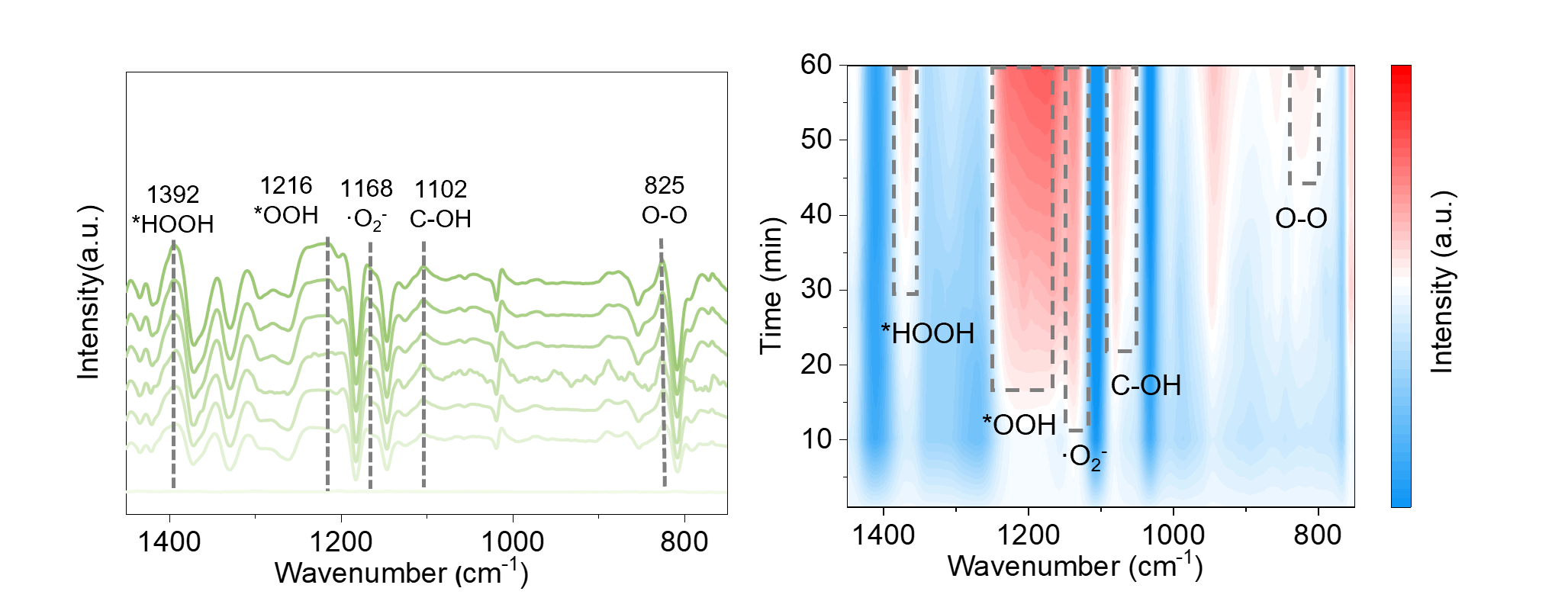


1. In situ DRIFTs spectrum (left) and infrared contour map (right) for COF-H in H_2_O_2_ photosynthesis.


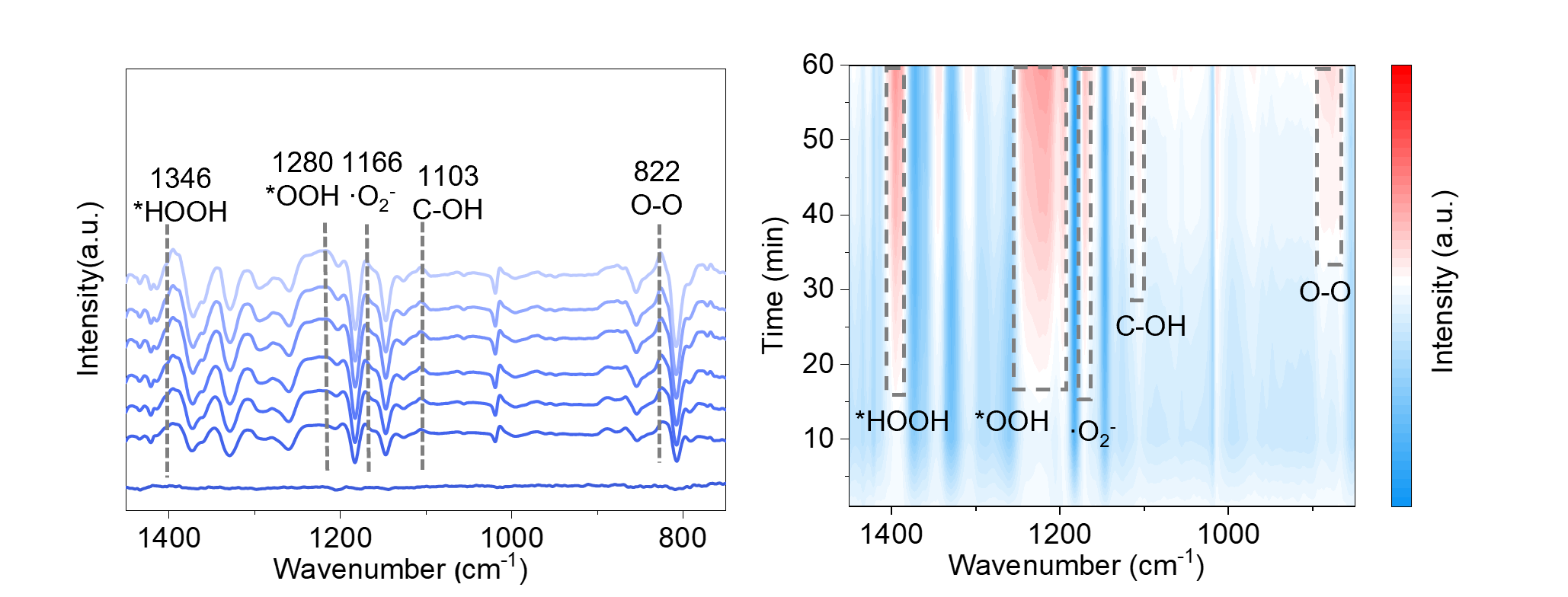


1. In situ DRIFTs spectrum (left) and infrared contour map (right) for COF-Br in H_2_O_2_ photosynthesis.


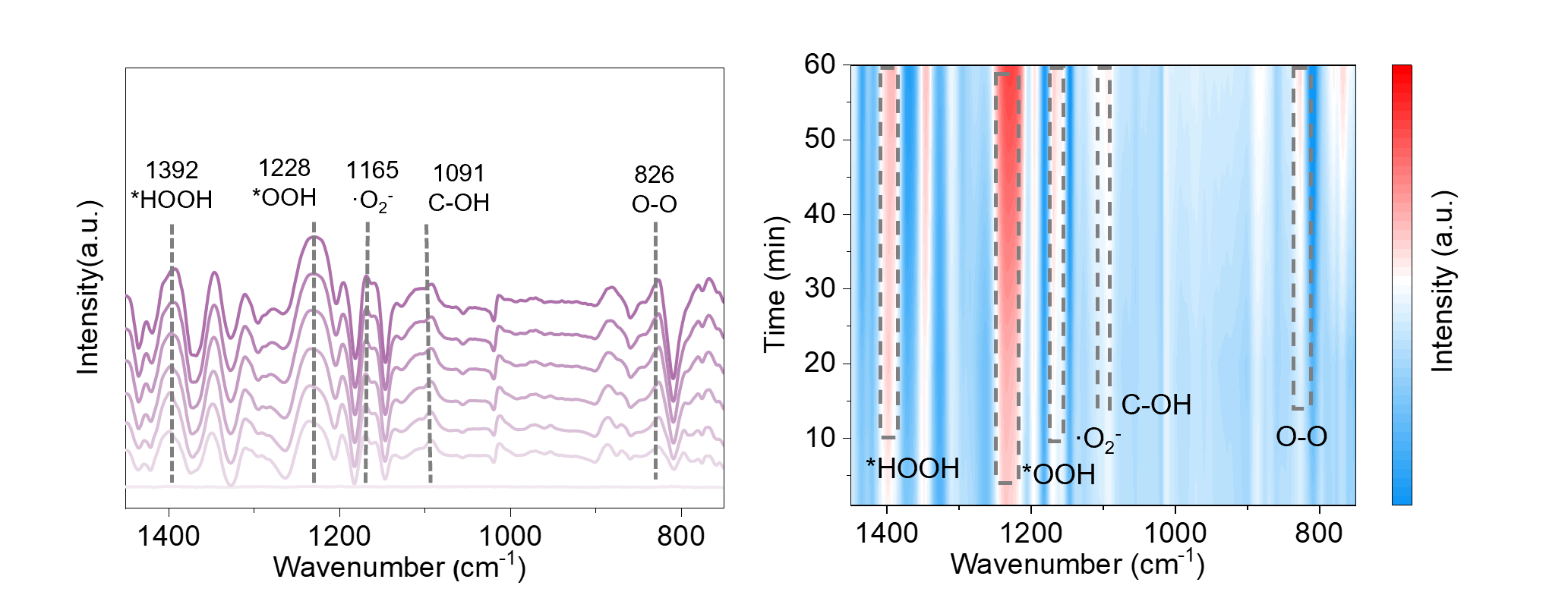


1. In situ DRIFTs spectrum (left) and infrared contour map (right) for COF-CN in H_2_O_2_ photosynthesis.


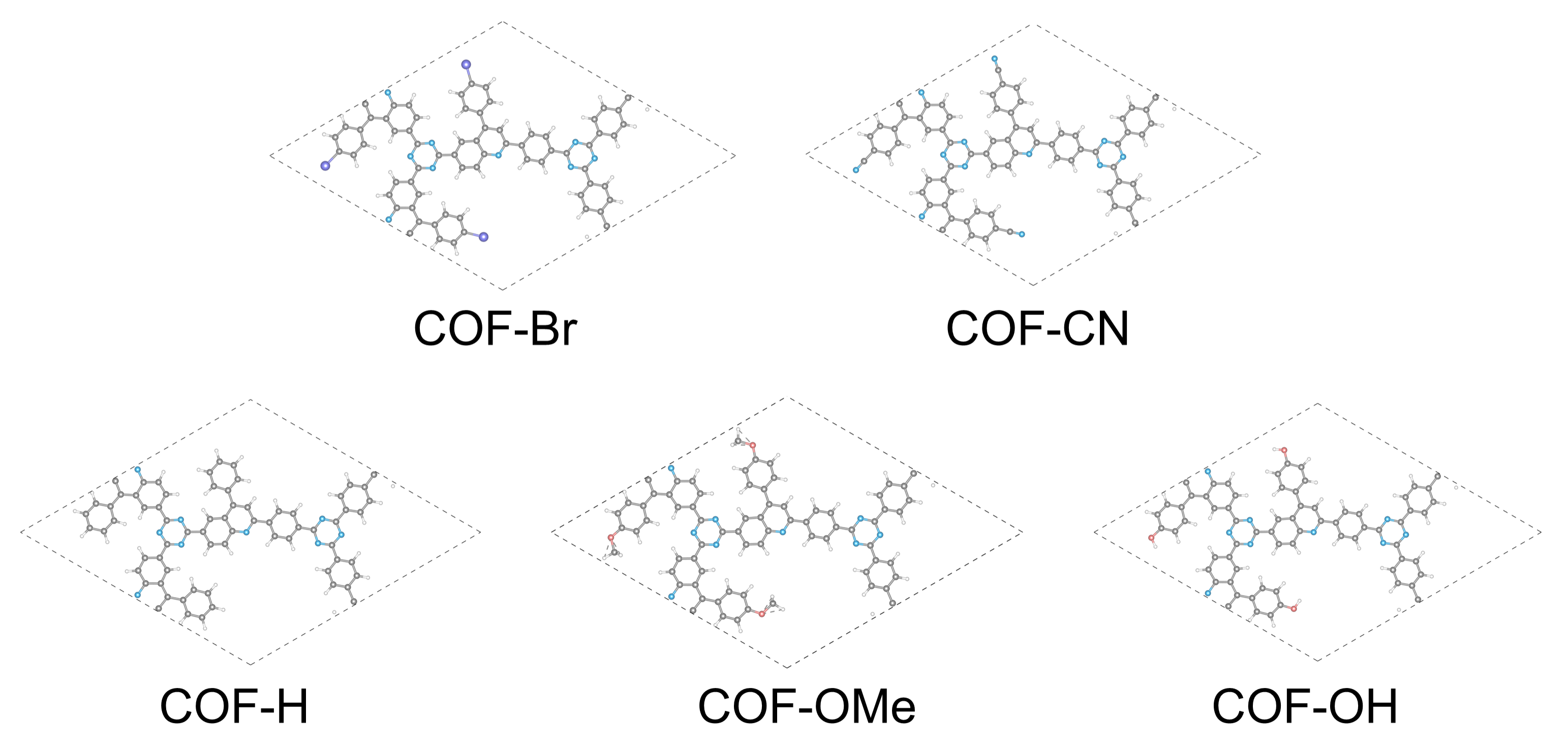


1. Structural models of COFs-R (-R=-OH, -OMe, -H, -Br and -CN).

**
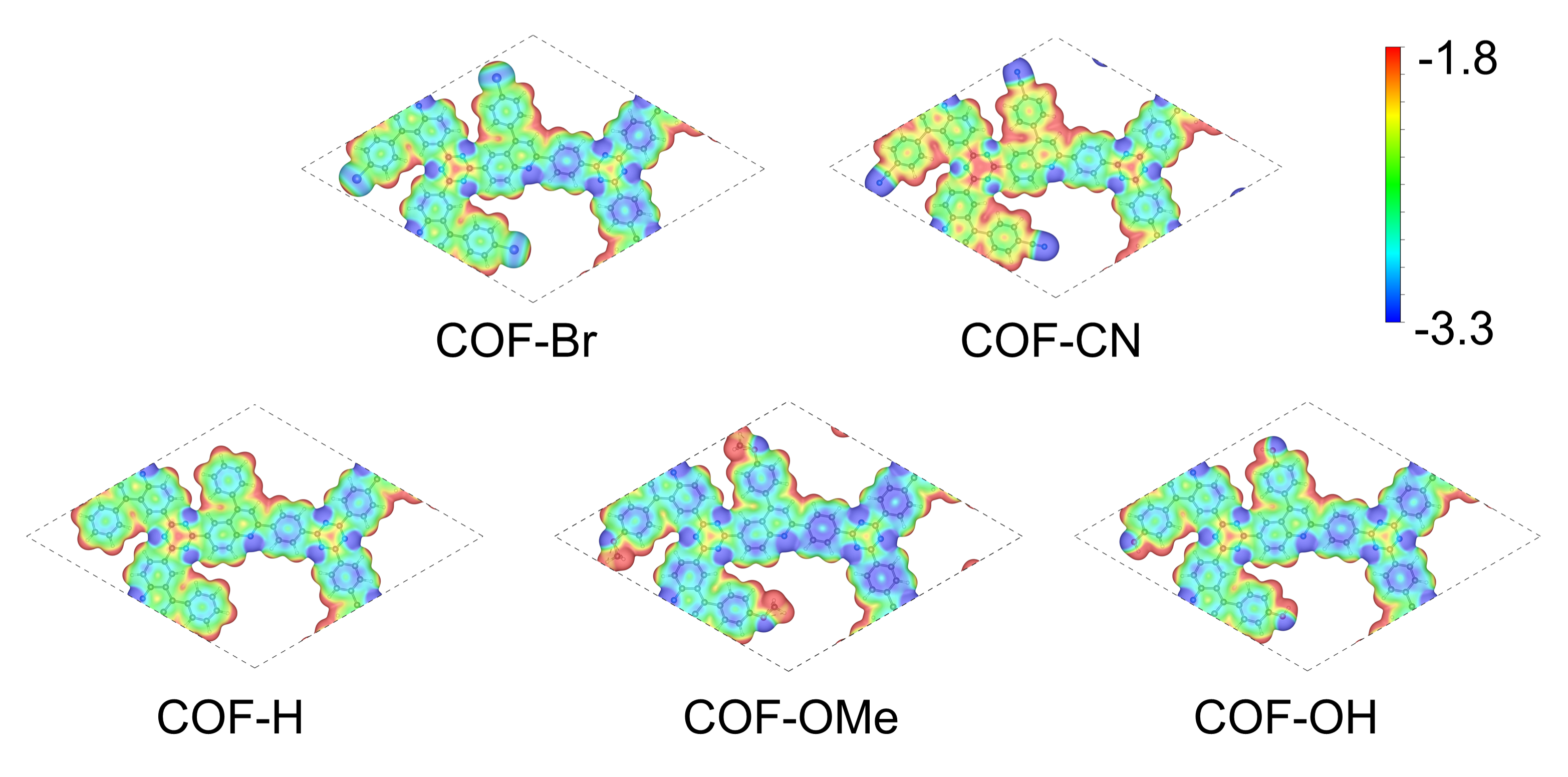
**

1. The electrostatic potential (ESP) of COFs-R, with blue indicating nucleophilic regions and red indicating electrophilic regions.


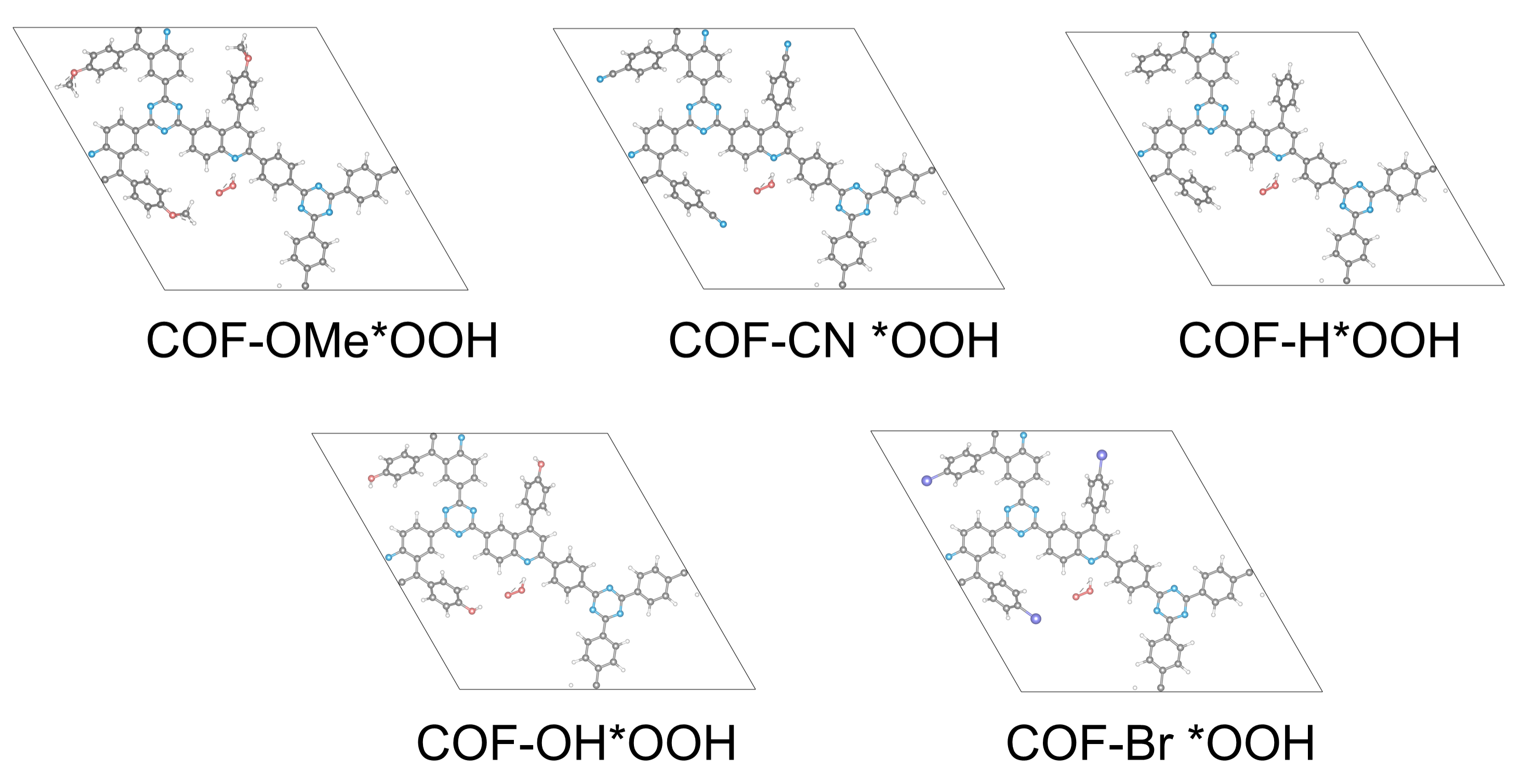


1. Structural models of COFs-R*OOH.


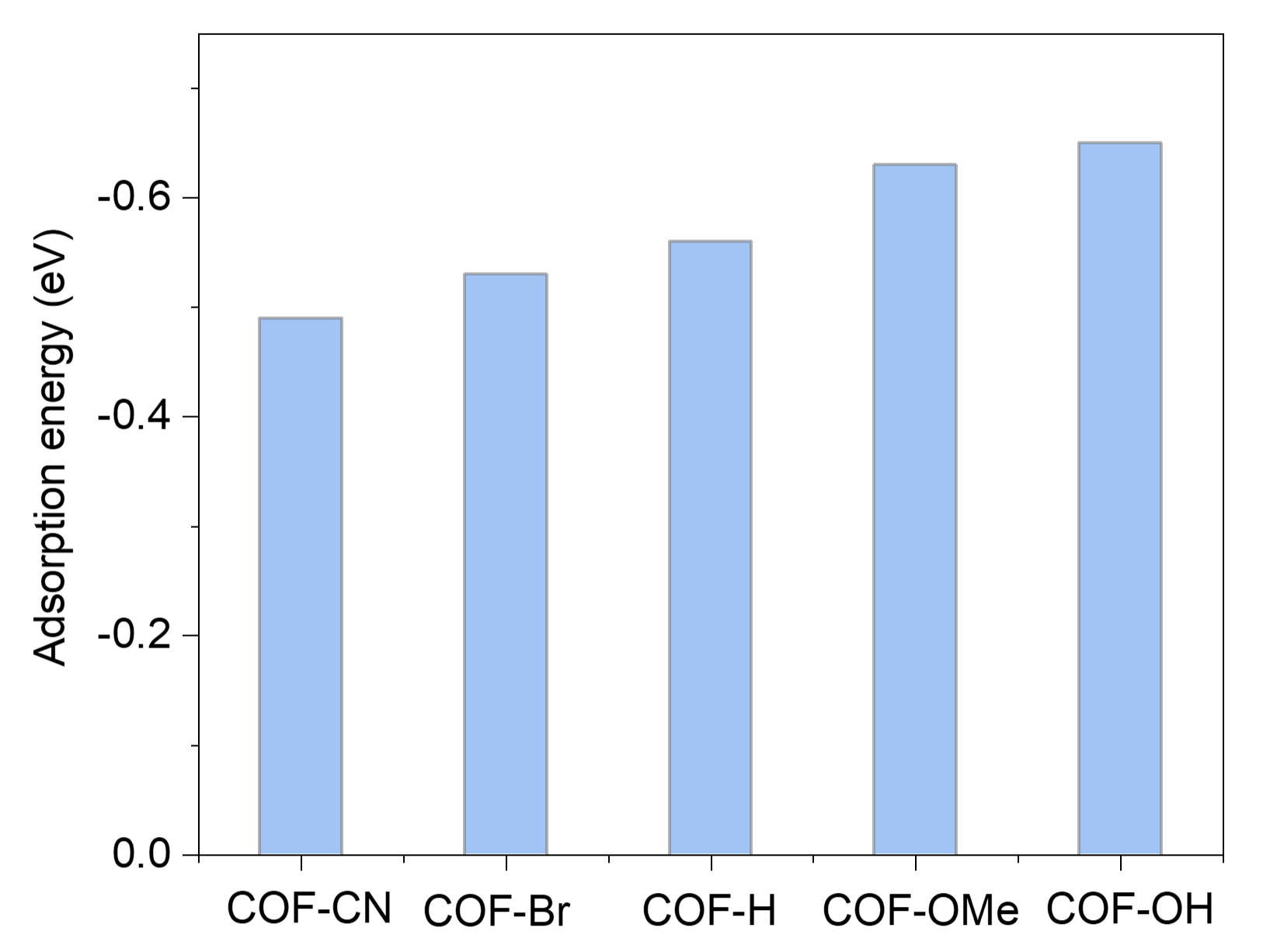


1. Adsorption energy of COFs-R*OOH.


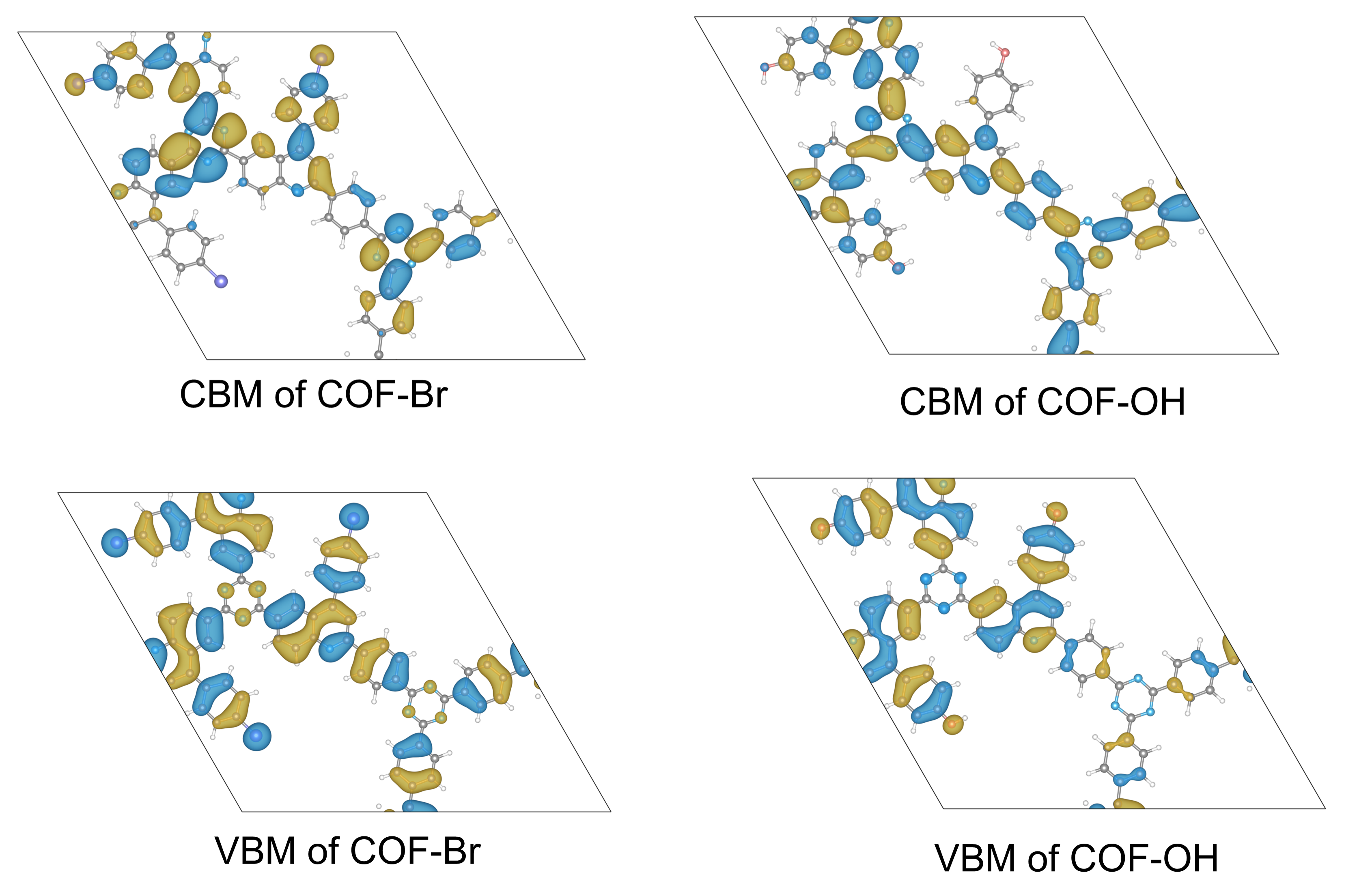


1. CBM/VBM of COF-Br and COF-OH.


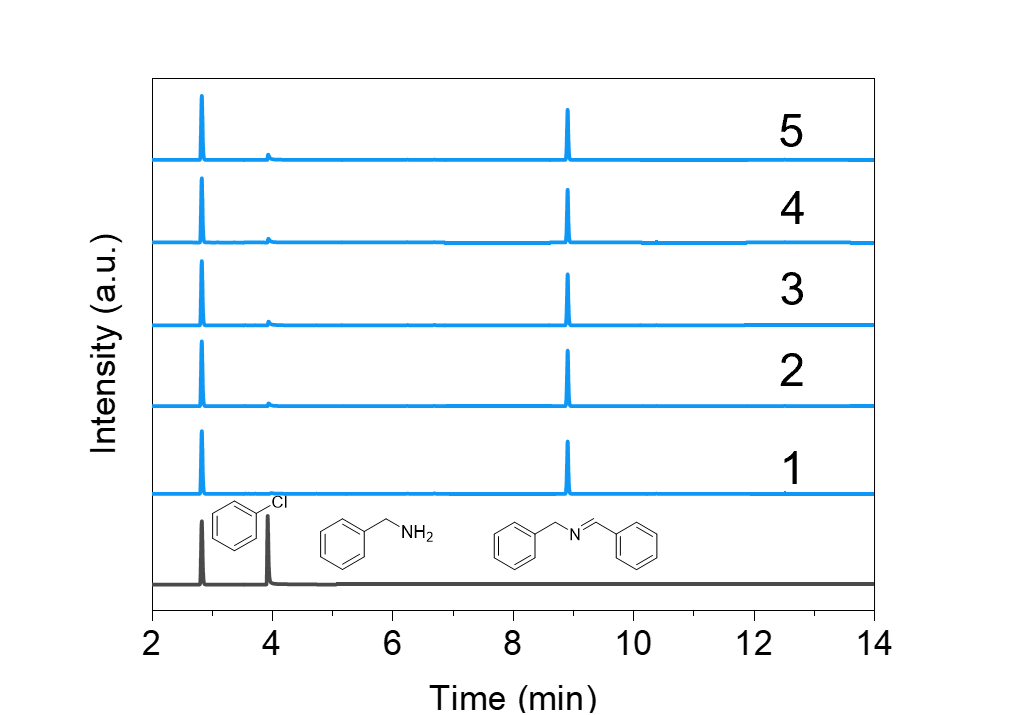


1. GC spectra of Benzylamine solution using COF-OMe as catalysts after multiple cycles.


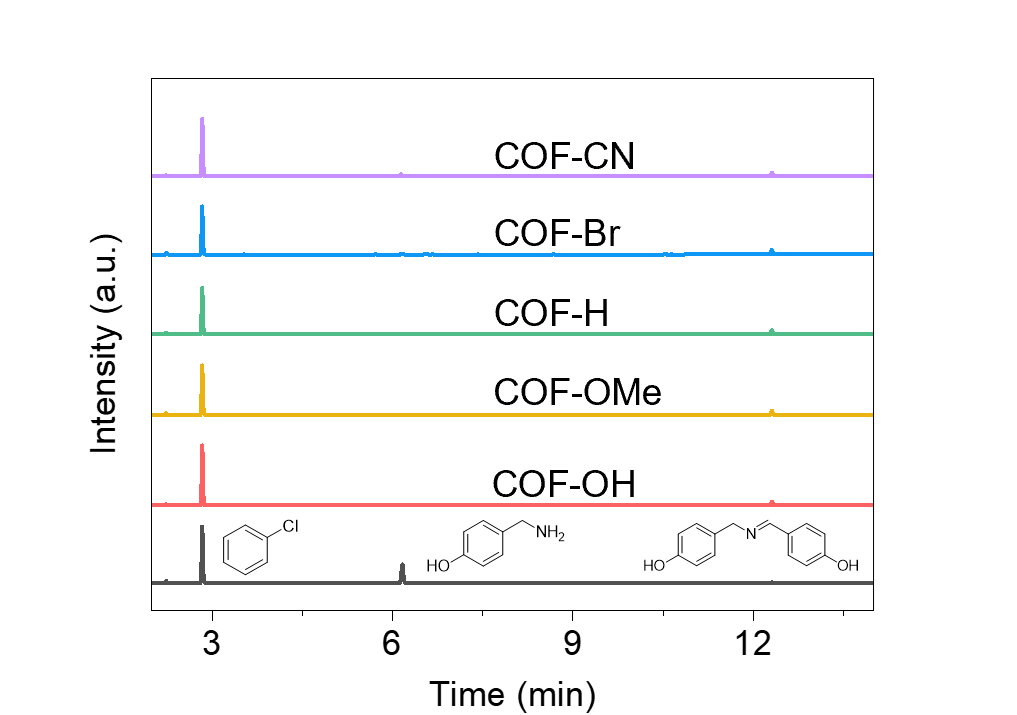


1. GC spectra of 4-Hydroxybenzylamine solution before and after the reaction using different COFs as catalysts.


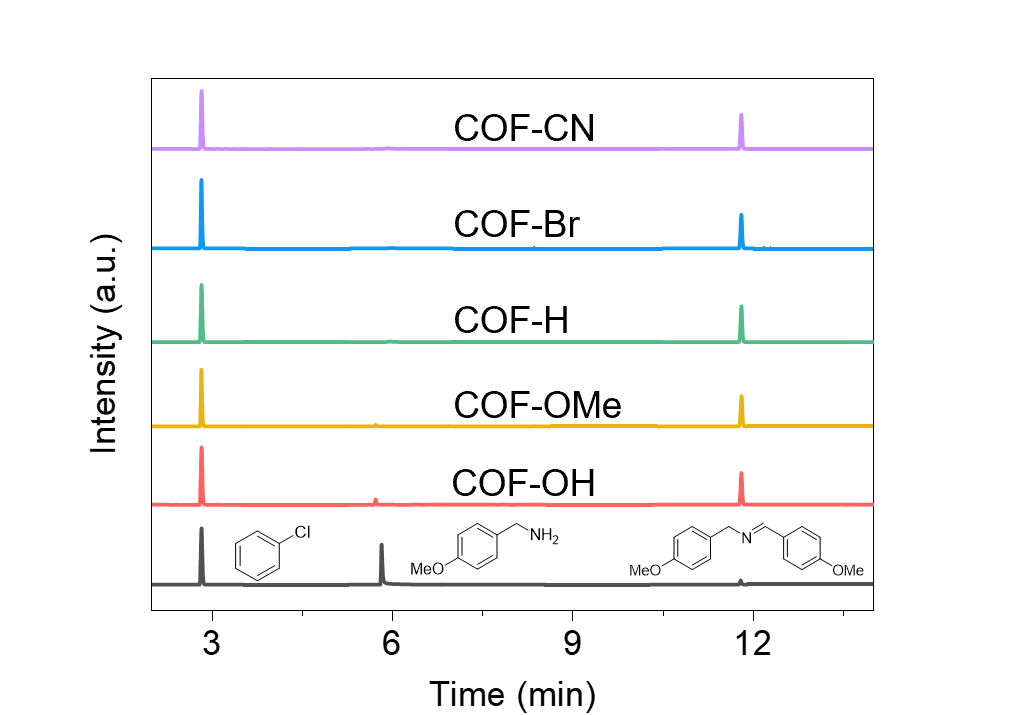


1. GC spectra of 4-Methoxybenzylamine solution before and after the reaction using different COFs as catalysts.


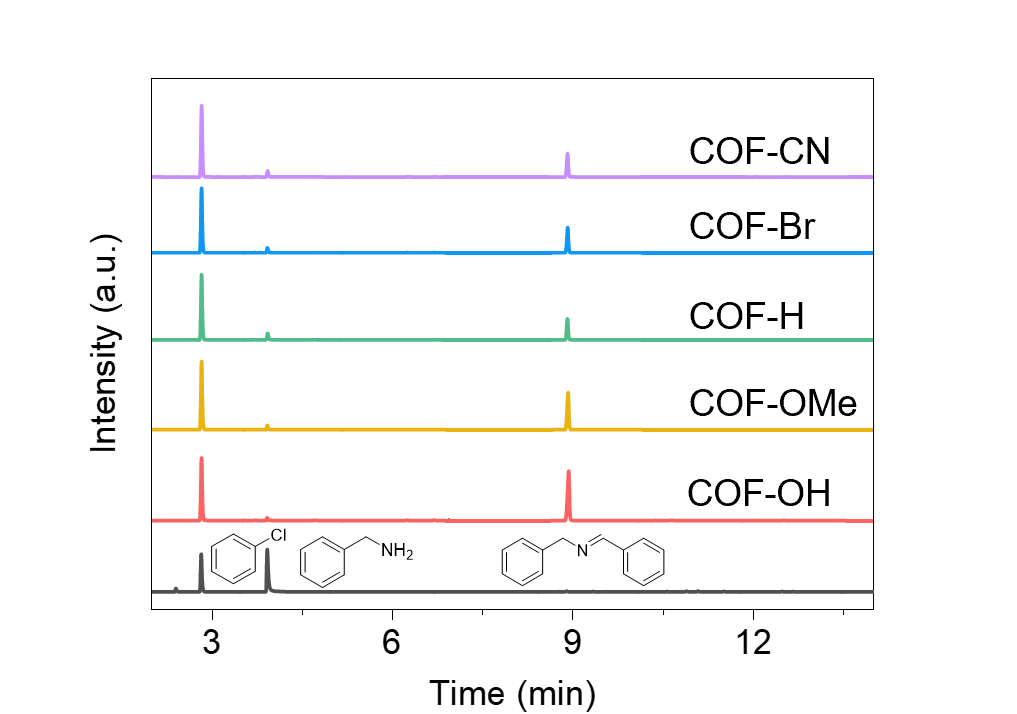


1. GC spectra of Benzylamine solution before and after the reaction using different COFs as catalysts.


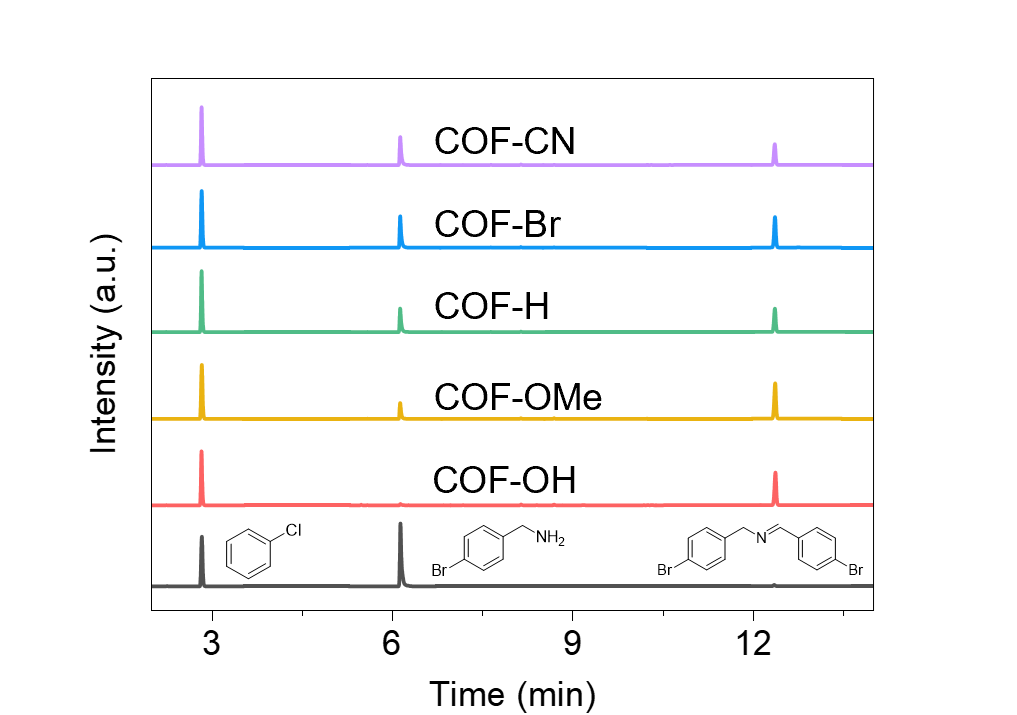


1. GC spectra of 4-Bromobenzylamine solution before and after the reaction using different COFs as catalysts.


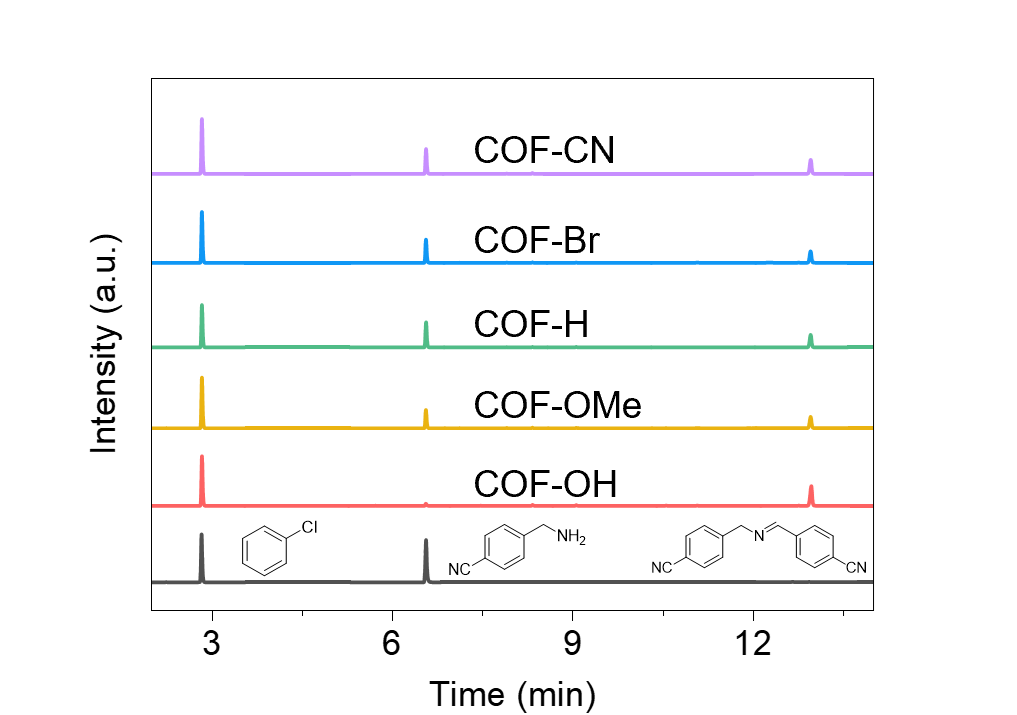


1. GC spectra of 4-Cyanobenzylamine solution before and after the reaction using different COFs as catalysts.


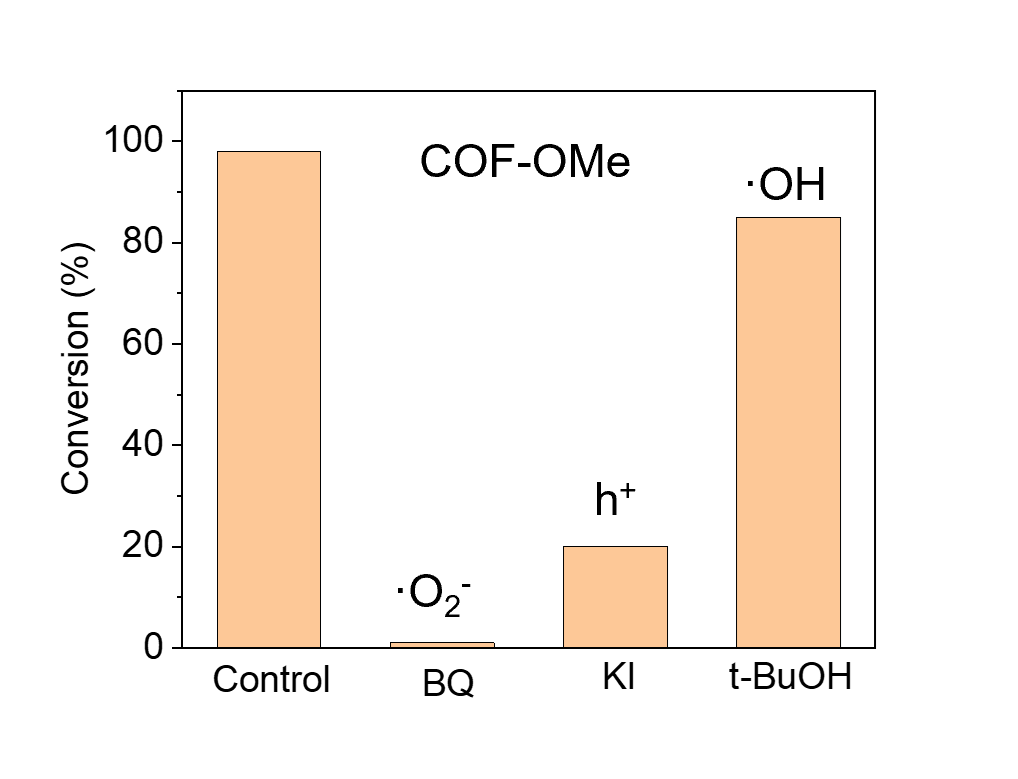


1. The benzylamine coupling reaction with different consuming reagents using COF-OMe as catalysts. (tert-butanol (t-BuOH, •OH scavenger), KI (h^+^ scavenger), benzoquinone (BQ, •O_2_^-^ scavenger)

| Publicationyear | Photocatalysts | Irradiation conditions /nm | H_2_O_2_ yields/ umol g^-1^ h^-1^ | Max AQY (%) | Ref. | |
| --- | --- | --- | --- | --- | --- | --- |
| 2025 | TBA-COF | λ> 420 nm | 8878 | 9.5% at 420 nm | ^[10]^ | |
| 2025 | MeO-QN-TA-COF | 460 nm, blue light | 7384 | 10.1% at 460 nm | | ^[11]^ |
| 2023 | TPdZ | λ> 420 nm | 7327 | 11.9 at 420 nm | ^[12]^ | |
| 2025 | EBBT-COF | λ> 400 nm | 5686 | 15.1% at 420nm | ^[13]^ | |
| 2025 | COF-OH | λ> 420 nm | 4458 | 7% at 400nm | This work | |
| 2024 | o-COFs | λ> 420 nm | 4396 | NR | ^[14]^ | |
| 2023 | Bpy-TAPT | λ> 420 nm | 4038 | 8.6% at 4.2 nm | ^[15]^ | |
| 2025 | β-TT-TDAN COF | 300 W Xe lamp | 3424 | [19.0% at 420 nm](mailto:19.02%25@420%20nm) | ^[16]^ | |
| 2024 | TT-COF-OH | λ> 400 nm | 3406 | 8.1% at 400 nm | ^[17]^ | |
| 2024 | FS-OHOMe-COF | λ≥420 nm | 2200 | 9.6% at 420 nm | ^[18]^ | |
| 2024 | COF-2CN | λ≥420 nm | 1601 | 6.8% at 459 nm | ^[19]^ | |
| 2025 | TFBP-DHBD | Xenon lamp | 1444 | NR | ^[20]^ | |
| 2023 | TaptBtt | 420-700 nm | 1407 | 4.6% at 450 nm | ^[2]^ | |
| 2023 | COF-NUST-16 | λ≥420 nm | 1081 | NR | ^[21]^ | |
| 2023 | COF-N32 | λ≥420 nm | 605 | 6.2% at 459 nm | ^[22]^ | |

1. Comparison of Photocatalytic Materials for H_2_O_2_ Production in pure water.
2. **Fractional Co-ordinates for COFs**

| **Im-COF_Pawley** | | | | |
| --- | --- | --- | --- | --- |
| **Space Group: P6̅ (174)**  **a = 23.44 Å, b = 23.44 Å, c = 3.19 Å**  **α = 90.0000 °, β = 90.0000 °, γ = 120.0000 °** | | | | |
| **Atom label** | **Atom type** | **x** | **y** | **z** |
| H1 | H | 5.426 | 13.159 | 0 |
| H2 | H | 7.039 | 12.047 | 0 |
| C3 | C | -1.062 | 12.953 | 0 |
| N4 | N | 0.039 | 12.374 | 0 |
| C5 | C | 2.452 | -12.726 | 0 |
| C6 | C | 3.444 | -13.543 | 0 |
| C7 | C | 4.649 | -13.111 | 0 |
| C8 | C | 4.901 | -11.841 | 0 |
| C9 | C | 3.894 | -11.029 | 0 |
| C10 | C | 2.692 | -11.464 | 0 |
| N11 | N | 6.302 | -10.097 | 0 |
| C12 | C | 6.177 | -11.322 | 0 |
| C13 | C | 8.516 | -10.206 | 0 |
| C14 | C | 7.394 | -9.526 | 0 |
| C15 | C | 7.37 | -8.247 | 0 |
| C16 | C | 8.444 | -7.577 | 0 |
| C17 | C | 9.568 | -8.181 | 0 |
| C18 | C | 9.591 | -9.472 | 0 |
| H19 | H | 3.292 | -14.524 | 0 |
| H20 | H | 5.359 | -13.797 | 0 |
| H21 | H | 4.029 | -10.047 | 0 |
| H22 | H | 1.949 | -10.806 | 0 |
| H23 | H | 6.502 | -7.764 | 0 |
| H24 | H | 8.39 | -6.586 | 0 |
| H25 | H | 10.472 | -9.823 | 0 |
| C26 | C | 10.833 | 7.611 | 0 |
| N27 | N | 10.623 | 6.385 | 0 |
|  |  |  |  |  |

| **COF-OH_Pawley** | | | | |
| --- | --- | --- | --- | --- |
|  | | | | |
| **Space Group: P3 (143)**  **a = 24.86 Å, b = 24.86 Å, c = 3.42 Å**  **α = 90.0000 °, β = 90.0000 °, γ = 120.0000 °** | | | | |
| **Atom label** | **Atom type** | **x** | **y** | **z** |
| H | H | -5.707 | -2.036 | -1.194 |
| O | O | -5.591 | -2.238 | -0.266 |
| C1 | C | 6.147 | 15.145 | 0.095 |
| C2 | C | 5.172 | 16.036 | -0.461 |
| C3 | C | 5.385 | 17.391 | -0.557 |
| C4 | C | 6.593 | 17.947 | -0.135 |
| C5 | C | 7.577 | 17.119 | 0.409 |
| C6 | C | 7.362 | 15.767 | 0.533 |
| H7 | H | 4.238 | 15.644 | -0.879 |
| H8 | H | 4.611 | 18.034 | -1.003 |
| H10 | H | 8.527 | 17.557 | 0.753 |
| H11 | H | 8.155 | 15.155 | 0.987 |
| C12 | C | 7.103 | 12.908 | -0.058 |
| C13 | C | 5.989 | 13.725 | 0.142 |
| H14 | H | 8.075 | 13.379 | -0.24 |
| C15 | C | -1.135 | 13.709 | 0.764 |
| N16 | N | -0.008 | 12.98 | 0.764 |
| C17 | C | 2.509 | -13.363 | -0.423 |
| C18 | C | 3.633 | -14.243 | -0.411 |
| C19 | C | 4.906 | -13.747 | -0.333 |
| C20 | C | 5.149 | -12.338 | -0.247 |
| C21 | C | 4.015 | -11.466 | -0.289 |
| C22 | C | 2.74 | -11.956 | -0.376 |
| N23 | N | 6.519 | -10.47 | 0.059 |
| C24 | C | 6.467 | -11.824 | -0.081 |
| C25 | C | 8.921 | -10.645 | 0.361 |
| C26 | C | 7.736 | -9.896 | 0.274 |
| C27 | C | 7.688 | -8.529 | 0.391 |
| C28 | C | 8.849 | -7.818 | 0.597 |
| C29 | C | 10.091 | -8.51 | 0.704 |
| C30 | C | 10.083 | -9.917 | 0.652 |
| H31 | H | 3.478 | -15.331 | -0.449 |
| H32 | H | 5.727 | -14.473 | -0.303 |
| H33 | H | 4.168 | -10.378 | -0.229 |
| H34 | H | 1.894 | -11.254 | -0.385 |
| H35 | H | 6.715 | -8.025 | 0.303 |
| H36 | H | 8.812 | -6.72 | 0.641 |
| H37 | H | 11.039 | -10.434 | 0.777 |
| C38 | C | 11.405 | 7.989 | -0.444 |
| N39 | N | 11.149 | 6.671 | -0.444 |

| **COF-OMe_Pawley** | | | | |
| --- | --- | --- | --- | --- |
| **Space Group: P3 (143)**  **a = 24.76 Å, b = 24.76 Å, c = 3.40 Å**  **α = 90.0000 °, β = 90.0000 °, γ = 120.0000 °** | | | | |
| **Atom label** | **Atom type** | **x** | **y** | **z** |
| C | C | -6.96 | -1.394 | 0.236 |
| O | O | -5.79 | -2.196 | 0.131 |
| C1 | C | 6.12 | 15.129 | 0.004 |
| C2 | C | 5.12 | 16.026 | -0.491 |
| C3 | C | 5.261 | 17.392 | -0.443 |
| C4 | C | 6.45 | 17.969 | 0.02 |
| C5 | C | 7.472 | 17.122 | 0.467 |
| C6 | C | 7.321 | 15.756 | 0.466 |
| H7 | H | 4.196 | 15.633 | -0.931 |
| H8 | H | 4.45 | 18.02 | -0.839 |
| H10 | H | 8.4 | 17.568 | 0.857 |
| H11 | H | 8.137 | 15.145 | 0.876 |
| C12 | C | 7.082 | 12.892 | -0.182 |
| C13 | C | 5.968 | 13.707 | 0.03 |
| H14 | H | 8.051 | 13.368 | -0.37 |
| C15 | C | -1.141 | 13.719 | 0.706 |
| N16 | N | -0.019 | 12.98 | 0.707 |
| C17 | C | 2.507 | -13.352 | -0.543 |
| C18 | C | 3.634 | -14.227 | -0.535 |
| C19 | C | 4.906 | -13.726 | -0.461 |
| C20 | C | 5.145 | -12.316 | -0.371 |
| C21 | C | 4.006 | -11.45 | -0.409 |
| C22 | C | 2.733 | -11.944 | -0.494 |
| N23 | N | 6.51 | -10.444 | -0.055 |
| C24 | C | 6.462 | -11.8 | -0.202 |
| C25 | C | 8.91 | -10.621 | 0.271 |
| C26 | C | 7.726 | -9.871 | 0.175 |
| C27 | C | 7.68 | -8.505 | 0.3 |
| C28 | C | 8.84 | -7.796 | 0.524 |
| C29 | C | 10.08 | -8.491 | 0.643 |
| C30 | C | 10.066 | -9.897 | 0.59 |
| H31 | H | 3.484 | -15.316 | -0.575 |
| H32 | H | 5.73 | -14.449 | -0.433 |
| H33 | H | 4.156 | -10.361 | -0.345 |
| H34 | H | 1.884 | -11.246 | -0.5 |
| H35 | H | 6.709 | -8 | 0.204 |
| H36 | H | 8.807 | -6.698 | 0.57 |
| H37 | H | 11.019 | -10.416 | 0.729 |
| C38 | C | 11.401 | 7.985 | -0.563 |
| N39 | N | 11.151 | 6.665 | -0.563 |
| H | H | -7.751 | -1.911 | 0.798 |
| H | H | -7.356 | -1.139 | -0.749 |
| H | H | -6.706 | -0.456 | 0.751 |

| **COF-H_Pawley** | | | | |
| --- | --- | --- | --- | --- |
| **Space Group: P3 (143)**  **a = 25.24Å, b = 25.24 Å, c = 3.49 Å**  **α = 90.0000 °, β = 90.0000 °, γ = 120.0000 °** | | | | |
| **Atom label** | **Atom type** | **x** | **y** | **z** |
| C1 | C | 6.474 | 15.35 | 0 |
| C2 | C | 5.555 | 16.447 | 0 |
| C3 | C | 5.939 | 17.774 | 0 |
| C4 | C | 7.283 | 18.129 | 0 |
| C5 | C | 8.234 | 17.115 | 0 |
| C6 | C | 7.846 | 15.791 | 0 |
| H7 | H | 4.477 | 16.366 | 0 |
| H8 | H | 5.172 | 18.562 | 0 |
| H9 | H | 7.584 | 19.184 | 0 |
| H10 | H | 9.302 | 17.373 | 0 |
| H11 | H | 8.702 | 15.11 | 0 |
| C12 | C | 7.212 | 13.025 | 0 |
| C13 | C | 6.144 | 13.922 | 0 |
| H14 | H | 8.238 | 13.38 | 0 |
| C15 | C | -1.134 | 13.91 | 0 |
| N16 | N | 0.009 | 13.198 | 0 |
| C17 | C | 2.509 | -13.537 | 0 |
| C18 | C | 3.646 | -14.4 | 0 |
| C19 | C | 4.92 | -13.896 | 0 |
| C20 | C | 5.182 | -12.485 | 0 |
| C21 | C | 4.034 | -11.63 | 0 |
| C22 | C | 2.754 | -12.127 | 0 |
| N23 | N | 6.626 | -10.615 | 0 |
| C24 | C | 6.524 | -11.967 | 0 |
| C25 | C | 9.076 | -10.851 | 0 |
| C26 | C | 7.893 | -10.079 | 0 |
| C27 | C | 7.863 | -8.706 | 0 |
| C28 | C | 9.03 | -7.976 | 0 |
| C29 | C | 10.279 | -8.657 | 0 |
| C30 | C | 10.25 | -10.071 | 0 |
| H31 | H | 3.502 | -15.491 | 0 |
| H32 | H | 5.731 | -14.634 | 0 |
| H33 | H | 4.186 | -10.539 | 0 |
| H34 | H | 1.906 | -11.425 | 0 |
| H35 | H | 6.884 | -8.207 | 0 |
| H36 | H | 8.986 | -6.878 | 0 |
| H37 | H | 11.239 | -10.502 | 0 |
| C38 | C | 11.575 | 8.086 | 0 |
| N39 | N | 11.346 | 6.759 | 0 |

| **COF-Br_Pawley** | | | | |
| --- | --- | --- | --- | --- |
| **Space Group: P3 (143)**  **a = 25.05Å, b = 25.05 Å, c = 3.43 Å**  **α = 90.0000 °, β = 90.0000 °, γ = 120.0000 °** | | | | |
| **Atom label** | **Atom type** | **x** | **y** | **z** |
| Br | Br | 6.842 | 19.921 | 3.554 |
| C1 | C | 6.182 | 15.173 | 0.031 |
| C2 | C | 5.211 | 16.072 | 0.588 |
| C3 | C | 5.402 | 17.432 | 0.59 |
| C4 | C | 6.584 | 18.003 | 0.095 |
| C5 | C | 7.573 | 17.146 | -0.409 |
| C6 | C | 7.388 | 15.785 | -0.453 |
| H7 | H | 4.294 | 15.686 | 1.047 |
| H8 | H | 4.618 | 18.075 | 1.015 |
| H10 | H | 8.51 | 17.564 | -0.805 |
| H11 | H | 8.176 | 15.165 | -0.904 |
| C12 | C | 7.12 | 12.925 | 0.133 |
| C13 | C | 6.008 | 13.755 | -0.02 |
| H14 | H | 8.099 | 13.387 | 0.302 |
| C15 | C | -1.145 | 13.828 | -0.56 |
| N16 | N | -0.022 | 13.088 | -0.56 |
| C17 | C | 2.501 | -13.431 | 0.285 |
| C18 | C | 3.638 | -14.293 | 0.292 |
| C19 | C | 4.908 | -13.783 | 0.259 |
| C20 | C | 5.146 | -12.369 | 0.201 |
| C21 | C | 3.998 | -11.515 | 0.208 |
| C22 | C | 2.725 | -12.02 | 0.251 |
| N23 | N | 6.54 | -10.49 | -0.014 |
| C24 | C | 6.473 | -11.846 | 0.106 |
| C25 | C | 8.957 | -10.675 | -0.23 |
| C26 | C | 7.774 | -9.92 | -0.172 |
| C27 | C | 7.749 | -8.551 | -0.27 |
| C28 | C | 8.924 | -7.847 | -0.426 |
| C29 | C | 10.166 | -8.544 | -0.516 |
| C30 | C | 10.135 | -9.953 | -0.483 |
| H31 | H | 3.497 | -15.384 | 0.319 |
| H32 | H | 5.734 | -14.503 | 0.25 |
| H33 | H | 4.142 | -10.424 | 0.165 |
| H34 | H | 1.872 | -11.326 | 0.244 |
| H35 | H | 6.778 | -8.036 | -0.207 |
| H36 | H | 8.896 | -6.748 | -0.459 |
| H37 | H | 11.089 | -10.477 | -0.6 |
| C38 | C | 11.484 | 8.029 | 0.293 |
| N39 | N | 11.251 | 6.705 | 0.293 |

| **COF-CN_Pawley** | | | | |
| --- | --- | --- | --- | --- |
| **Space Group: P3 (143)**  **a = 25.34Å, b = 25.34 Å, c = 3.33 Å**  **α = 90.0000 °, β = 90.0000 °, γ = 120.0000 °** | | | | |
| **Atom label** | **Atom type** | **x** | **y** | **z** |
| C | C | 6.975 | 19.48 | 0.24 |
| N | N | 7.128 | 20.628 | 0.285 |
| C1 | C | 6.273 | 15.236 | 0.089 |
| C2 | C | 5.312 | 16.157 | 0.626 |
| C3 | C | 5.533 | 17.513 | 0.642 |
| C4 | C | 6.749 | 18.051 | 0.196 |
| C5 | C | 7.739 | 17.176 | -0.278 |
| C6 | C | 7.511 | 15.822 | -0.347 |
| H7 | H | 4.374 | 15.795 | 1.064 |
| H8 | H | 4.75 | 18.175 | 1.041 |
| H10 | H | 8.706 | 17.57 | -0.626 |
| H11 | H | 8.301 | 15.189 | -0.777 |
| C12 | C | 7.161 | 12.967 | 0.116 |
| C13 | C | 6.061 | 13.822 | 0.009 |
| H14 | H | 8.151 | 13.405 | 0.282 |
| C15 | C | -1.156 | 14.001 | -0.422 |
| N16 | N | -0.031 | 13.255 | -0.421 |
| C17 | C | 2.494 | -13.542 | 0.093 |
| C18 | C | 3.653 | -14.378 | 0.106 |
| C19 | C | 4.918 | -13.848 | 0.101 |
| C20 | C | 5.155 | -12.431 | 0.071 |
| C21 | C | 3.987 | -11.601 | 0.06 |
| C22 | C | 2.716 | -12.126 | 0.074 |
| N23 | N | 6.591 | -10.541 | -0.063 |
| C24 | C | 6.492 | -11.898 | 0.039 |
| C25 | C | 9.026 | -10.751 | -0.186 |
| C26 | C | 7.848 | -9.985 | -0.159 |
| C27 | C | 7.855 | -8.614 | -0.228 |
| C28 | C | 9.047 | -7.92 | -0.325 |
| C29 | C | 10.289 | -8.623 | -0.394 |
| C30 | C | 10.225 | -10.035 | -0.389 |
| H31 | H | 3.532 | -15.472 | 0.12 |
| H32 | H | 5.747 | -14.565 | 0.1 |
| H33 | H | 4.115 | -10.507 | 0.038 |
| H34 | H | 1.856 | -11.44 | 0.063 |
| H35 | H | 6.889 | -8.087 | -0.191 |
| H36 | H | 9.028 | -6.821 | -0.339 |
| H37 | H | 11.176 | -10.565 | -0.501 |
| C38 | C | 11.607 | 8.096 | 0.094 |
| N39 | N | 11.407 | 6.761 | 0.094 |

1. XRD peak positions corresponding to different crystal planes of COFs.

| **_Samples_ ^Peaks^** | **(100)** | **(110)** | **(200)** | **(210)** | **(220)** | **(001)** |
| --- | --- | --- | --- | --- | --- | --- |
| Im-COF | 4.1° | 7.0° | 8.1° | 10.8° | 14.6° | 25.9° |
| COF-OH | 4.0° | 7.0° | 8.0° | 10.7° | 14.6° | 25.8° |
| COF-OMe | 4.0° | 7.0° | 8.1° | 10.7° | 14.6° | 25.8° |
| COF-H | 4.0° | 7.0° | 8.0° | 10.6° | 14.5° | 25.6° |
| COF-Br | 4.0° | 7.0° | 8.0° | 10.6° | 14.6° | 25.6° |
| COF-CN | 4.0° | 7.0° | 8.0° | 10.7° | 14.7° | 25.8° |

1. Unit cell parameters of Im-COF and COFs-R.

|  | Im-COF | COF-OH | COF-OMe | COF-H | COF-Br | COF-CN |
| --- | --- | --- | --- | --- | --- | --- |
| a | 23.44 Å | 24.86 Å | 24.76 Å | 25.24 Å | 25.05 Å | 25.34 Å |
| b | 23.44 Å | 24.86 Å | 24.76 Å | 25.24 Å | 25.05 Å | 25.34 Å |
| c | 3.19 Å | 3.42 Å | 3.40 Å | 3.49 Å | 3.43 Å | 3.33 Å |
| α | 90 ° | 90 ° | 90 ° | 90 ° | 90 ° | 90 ° |
| β | 90 ° | 90 ° | 90 ° | 90 ° | 90 ° | 90 ° |
| γ | 120 ° | 120 ° | 120 ° | 120 ° | 120 ° | 120 ° |

1. Elemental analysis of COFs

|  | C (wt%) | N (wt%) | H (wt%) |
| --- | --- | --- | --- |
| Im-COF | 74.82 | 18.08 | 4.16 |
| COF-OH | 63.86 | 11.08 | 5.13 |
| COF-OMe | 58.82 | 12.04 | 5.06 |
| COF-H | 57.86 | 13.51 | 4.99 |
| COF-Br | 63.17 | 14.51 | 4.42 |
| COF-CN | 52.41 | 11.91 | 5.09 |

1. Photocatalytic H_2_O_2_ production performance of Im-COF and COFs-R

| **Samples** | **H_2_O_2_ yield rate (μmol g^-1^ h^-1^)** | **Irradiated conditions (300W Xe lamp)** | **AQY (%)** | **SCC (%)** | **Solvent (ml)** |
| --- | --- | --- | --- | --- | --- |
| **Im-COF** | 2795 | λ ≥ 420 nm (12.5cm^2^, 300 mWcm^-2^) |  |  | Water (20) |
|  | 4074 |  |  |  | Water: BA (18:2) |
| **COF-OH** | 4456 |  |  |  | Water (20) |
|  | 5284 |  |  | 0.15 | Water: BA (18:2) |
| **COF-OMe** | 4138 |  |  |  | Water (20) |
|  | 5497 |  |  | 0.16 | Water: BA (18:2) |
|  |  | λ = 400 nm (12.5cm^2^, 30 mWcm^-2^) | 4 |  | Water (40) |
|  |  |  | 7 |  | Water: BA (36:4) |
| **COF-H** | 3224 | λ ≥ 420 nm (12.5cm^2^, 300 mWcm^-2^) |  |  | Water (20) |
|  | 4500 |  |  |  | Water: BA (18:2) |
| **COF-Br** | 2125 |  |  |  | Water (20) |
|  | 3812 |  |  |  | Water: BA (18:2) |
| **COF-CN** | 2032 |  |  |  | Water (20) |
|  | 2788 |  |  |  | Water: BA (18:2) |

**Reference**

[1] P. Das, J. Roeser, A. Thomas, Solar Light Driven H_2_O_2_ Production and Selective Oxidations Using a Covalent Organic Framework Photocatalyst Prepared by a Multicomponent Reaction, *Angew. Chem. Int. Ed.* **2023**, 62, e202304349.

[2] C. Qin, X. Wu, L. Tang, X. Chen, M. Li, Y. Mou, B. Su, S. Wang, C. Feng, J. Liu, X. Yuan, Y. Zhao, H. Wang, Dual donor-acceptor covalent organic frameworks for hydrogen peroxide photosynthesis, *Nat. Commun.* **2023**, 14.

[3] Z. Wei, M. Liu, Z. Zhang, W. Yao, H. Tan, Y. Zhu, Efficient visible-light-driven selective oxygen reduction to hydrogen peroxide by oxygen-enriched graphitic carbon nitride polymers, *Energy Environ. Sci.* **2018**, 11, 2581.

[4] Y. Shiraishi, T. Takii, T. Hagi, S. Mori, Y. Kofuji, Y. Kitagawa, S. Tanaka, S. Ichikawa, T. Hirai, Resorcinol–formaldehyde resins as metal-free semiconductor photocatalysts for solar-to-hydrogen peroxide energy conversion, *Nat. Mater.* **2019**, 18, 985.

[5] C. Yang, S. Wan, B. Zhu, J. Yu, S. Cao, Calcination-regulated Microstructures of Donor-Acceptor Polymers towards Enhanced and Stable Photocatalytic H_2_O_2_ Production in Pure Water, *Angew. Chem. Int. Ed.* **2022**, 61, e202208438.

[6] P. E. Blöchl, Projector augmented-wave method, *Phys. Rev. B* **1994**, 50, 17953.

[7] J. P. Perdew, K. Burke, M. Ernzerhof, Generalized Gradient Approximation Made Simple, *Phys. Rev. Lett.* **1996**, 77, 3865.

[8] G. Henkelman, A. Arnaldsson, H. Jónsson, A fast and robust algorithm for Bader decomposition of charge density, *Comput. Mater. Sci* **2006**, 36, 354.

[9] V. Wang, N. Xu, J.-C. Liu, G. Tang, W.-T. Geng, VASPKIT: A user-friendly interface facilitating high-throughput computing and analysis using VASP code, *Comput. Phys. Commun.* **2021**, 267, 108033.

[10] J.-Y. Yue, Z.-X. Pan, R.-Z. Zhang, Q. Xu, P. Yang, B. Tang, One-Pot Synthesis of Fully Conjugated Covalent Organic Frameworks via the Pictet–Spengler Reaction for Boosting H_2_O_2_ Photogeneration in Real Seawater, *Adv. Funct. Mater.* **2025**, 2421514.

[11] R. Liu, M. Zhang, F. Zhang, B. Zeng, X. Li, Z. Guo, X. Lang, Linkage Microenvironment Modulation in Triazine‐Based Covalent Organic Frameworks for Enhanced Photocatalytic Hydrogen Peroxide Production, *Small* **2025**, 2411625.

[12] Q. Liao, Q. Sun, H. Xu, Y. Wang, Y. Xu, Z. Li, J. Hu, D. Wang, H. Li, K. Xi, Regulating Relative Nitrogen Locations of Diazine Functionalized Covalent Organic Frameworks for Overall H_2_O_2_ Photosynthesis, *Angew. Chem. Int. Ed.* **2023**, 62, e202310556.

[13] B. Li, J. Chen, K. Wang, D. Qi, T. Wang, J. Jiang, Ethynyl-Linked Donor–Acceptor Covalent Organic Framework for Highly Efficient Photocatalytic H_2_O_2_ Production, *Adv. Energy Mater.* **2025**, 2404497.

[14] T. Yang, D. Zhang, A. Kong, Y. Zou, L. Yuan, C. Liu, S. Luo, G. Wei, C. Yu, Robust Covalent Organic Framework Photocatalysts for H_2_O_2_ Production: Linkage Position Matters, *Angew. Chem. Int. Ed.* **2024**, 63, e202404077.

[15] Y. Liu, W.-K. Han, W. Chi, Y. Mao, Y. Jiang, X. Yan, Z.-G. Gu, Substoichiometric covalent organic frameworks with uncondensed aldehyde for highly efficient hydrogen peroxide photosynthesis in pure water, *Appl. Catal. B-Environ.* **2023**, 331, 122691.

[16] W. Zhang, M. Sun, J. Cheng, X. Wu, H. Xu, Regulating Electron Distribution in Regioisomeric Covalent Organic Frameworks for Efficient Solar-Driven Hydrogen Peroxide Production, *Adv. Mater.*, 37, 2500913.

[17] Y. Yao, C. Zhu, R. Liu, Q. Fang, S. Song, B. Chen, Y. Shen, Synergistic Tri-efficiency Enhancement Utilizing Functionalized Covalent Organic Frameworks for Photocatalytic H_2_O_2_ Production, *Small* **2024**, 20, 2404885.

[18] C. Shu, X. Yang, L. Liu, X. Hu, R. Sun, X. Yang, A. I. Cooper, B. Tan, X. Wang, Mixed-Linker Strategy for the Construction of Sulfone-Containing D–A–A Covalent Organic Frameworks for Efficient Photocatalytic Hydrogen Peroxide Production, *Angew. Chem. Int. Ed.* **2024**, 63, e202403926.

[19] Y. Hou, P. Zhou, F. Liu, Y. Lu, H. Tan, Z. Li, M. Tong, J. Ni, Efficient Photosynthesis of Hydrogen Peroxide by Cyano-Containing Covalent Organic Frameworks from Water, Air and Sunlight, *Angew. Chem. Int. Ed.* **2024**, 63, e202318562.

[20] C. Sun, Y. Han, H. Guo, R. Zhao, Y. Liu, Z. Lin, Z. Xiao, Z. Sun, M. Luo, S. Guo, Proton Reservoir in Covalent Organic Framework Compensating Oxygen Reduction Reaction Enhances Hydrogen Peroxide Photosynthesis, *Adv. Mater.* **2025**, 2502990.

[21] C.-H. Hsueh, C. He, J. Zhang, X. Tan, H. Zhu, W.-C. M. Cheong, A.-Z. Li, X. Chen, H. Duan, Y. Zhao, C. Chen, Three-Dimensional Mesoporous Covalent Organic Framework for Photocatalytic Oxidative Dehydrogenation to Quinoline, *J. Am. Chem. Soc.* **2024**, 146, 33857.

[22] F. Liu, P. Zhou, Y. Hou, H. Tan, Y. Liang, J. Liang, Q. Zhang, S. Guo, M. Tong, J. Ni, Covalent organic frameworks for direct photosynthesis of hydrogen peroxide from water, air and sunlight, *Nat. Commun.* **2023**, 14.
